# Single-Cell RNA Sequencing Reveals Unique Alterations in the Immune Panorama and Treg Subpopulations in Mice during the Late Stages of Echinococcus granulosus Infection

**DOI:** 10.1128/iai.00029-23

**Published:** 2023-04-11

**Authors:** Jianwen Wu, Jing Xiao, Min Bai, Chunli Shi, Yunzhuo Xin, Wei Zhao, Xiaoping Gao, Mei Yin, Jiaqing Zhao

**Affiliations:** a School of Basic Medicine, Ningxia Medical University, Yinchuan, China; b Research Center for Medical Science and Technology, Ningxia Medical University, Yinchuan, China; c Ningxia Institute of Medical Science, Yinchuan, China; d Ningxia Key Laboratory of Prevention and Control of Common Infectious Diseases, Yinchuan, China; e Department of Respiratory Medicine, General Hospital of Ningxia Medical University, Yinchuan, China; f Department of Otolaryngology Head and Neck Surgery, General Hospital of Ningxia Medical University, Yinchuan, China; g Department of Molecular Biology, Shanghai Centre for Clinical Laboratory, Shanghai, China; h Shiyan Integrated Traditional Chinese and Western Medicine Hospital, Shiyan, China; University of Pennsylvania

**Keywords:** single-cell RNA sequencing, single-cell TCR sequencing, single-cell BCR sequencing, *Echinococcus granulosus*, immune cells, Tregs

## Abstract

Cystic echinococcosis (CE) is a common zoonotic parasitic disease that seriously impacts public health. However, the full spectrum of immune cell changes in *Echinococcus granulosus* infection, especially the negative immune regulation of subpopulations of regulatory T (Treg) cells, are not yet well understood. In this study, we used single-cell RNA sequencing and immunome repertoire (IR) sequencing to analyze 53,298 cells from the spleens and peripheral blood mononuclear cells (PBMCs) of healthy and *E. granulosus*-infected mice. We used immunofluorescence combined with RNA fluorescence *in situ* hybridization and quantitative real-time PCR to verify the sequencing results. Our results showed tissue-specific immune system alterations in mice infected with *E. granulosus*. *E. granulosus*-infected mice induced a subpopulation of CD4^+^ cells with type I interferon production potential. Furthermore, there were six different Treg cell subpopulations *in vivo* at three stages of differentiation, and Treg subpopulations of different classes and different stages of differentiation showed tissue specificity. After infection, the Lag3^hi^ Treg and *Gpr83*^+^*Igfbp4*^+^ naive Treg subpopulations were specifically induced in PBMCs and the spleen, respectively. Furthermore, T follicular helper 2 (Tfh2) cells with high expression of *Cxxc5* and *Spock2* were found in *E. granulosus*-infected mice. Our data uncovered changes in the full spectrum of immune cells in mice following the late stages of *E. granulosus* infection, including subpopulations of cells that have not been emphasized in previous studies. These results further enrich the study of the bidirectional immunomodulatory mechanism and offer a different perspective for subsequent studies of infection in *E. granulosus*.

## INTRODUCTION

Cystic echinococcosis (CE) is a common zoonotic parasitic disease in which the eggs of *Echinococcus granulosus* are the source of infection. When mistakenly ingested by humans, *E. granulosus* can cause damage to multiple organs and threaten the life of the host (1). CE is 1 of 17 neglected tropical diseases recognized by the World Health Organization that affects over 1 million people and is responsible for over $3 billion dollars in expenses every year. This parasitic disease causes considerable human morbidity and mortality and is difficult to diagnose, treat, and control (2, 3). As a result, CE has become a worldwide public health concern; it is often a lifelong condition, and the medical costs borne by countries of endemicity are high (4, 5). In recent years, great progress has been made in the diagnosis, treatment, and development of vaccines (6–8). For example, in conjunction with albendazole, the use of metal nanoparticle composites is facilitating the treatment of CE (9). Moreover, researchers have developed vaccines that are expected to prevent *E. granulosus* infection, such as recombinant antigens EgP-29 and glutathione *S*-transferases (10, 11).

Although some progress has been made in the study of CE, changes in the full spectrum of immune cells, immunomodulatory mechanisms, early diagnosis, and optimal treatment still need further research (12). *E. granulosus* does not reproduce as adults in the host but produces eggs or larvae that can infect new hosts. Furthermore, *E. granulosus* tends to cause a stable chronic infection in the body, inducing an immune escape response resulting in the parasite living in the host for a long time (13). Faster growth of cysts in CE patients with AIDS suggests that immunosuppression may play a role in CE progression (14). Similar to human CE patients, infection of mice by *E. granulosus* induced a predominant T helper 1 (Th1)-type immune response in the early stages, while gradually shifting to a Th2-type response in the middle and late stages of infection (15). Experimental and clinical studies of CE infections have shown that Th1-related immune responses are associated with protection, and Th2-related immune responses are associated with parasite growth (16).

Several studies have revealed that immunosuppressive cells, such as regulatory T (Treg) cells, regulatory B (Breg) cells, and myeloid-derived suppressor cells (MDSCs), begin to proliferate in the late stage of *E. granulosus* infection (17–19). T cells from asymptomatic carriers of helminth infection exhibit a distorted cytokine expression profile. Preferring interleukin-4 (IL-4) over IL-17 and interferon-γ (IFN-γ) and containing a more pronounced IL-10 and transforming growth factor-β (TGF-β) component, a strong association has emerged between long-term helminth infection and Treg cell activity (20).Treg cells are considered to be powerful negative regulators of the inflammatory response, and different types of Treg cells may play specific roles in different disease models (21). However, the mechanisms of Treg cell proliferation in CE are not fully resolved, and the molecular program of Treg cell development and heterogeneity in each population are not clear at least, in part, due to the lack of comprehensive analysis of single cells.

The spleen and peripheral blood mononuclear cells (PBMCs) harbor a pool of immune cells with largely unexplored phenotypes and development. Therefore, based on the 10x Genomics platform, single-cell RNA sequencing (scRNA-seq) and immune repertoire (IR) sequencing technologies were used to examine spleens and PBMCs from BALB/c mice receiving protoscoleces or phosphate-buffered saline (PBS). We explored the dynamics of circulating immune cells at single-cell resolution in mice infected with *E. granulosus* and provide preliminary insights into the immune response during the progression of CE. Additionally, our deep excavation of the Treg cell populations with high *Foxp3* expression revealed that Treg cells were composed of six subpopulations with different transcriptomes and multifaceted characteristics, a finding that adds to our understanding of the cellular heterogeneity of Treg cells produced by late *E. granulosus* infection. Additionally, Treg cell subpopulation six was substantially increased in PBMCs and spleens of mice after infection, and its specific highly expressed genes *Gpr83* and *Igfbp4* have the potential to become biomarkers for the diagnosis and prognosis of CE.

## RESULTS

### scRNA-seq revealed tissue-common and tissue-specific immune system alterations in mice infected with *E. granulosus*.

**FIG 1:**
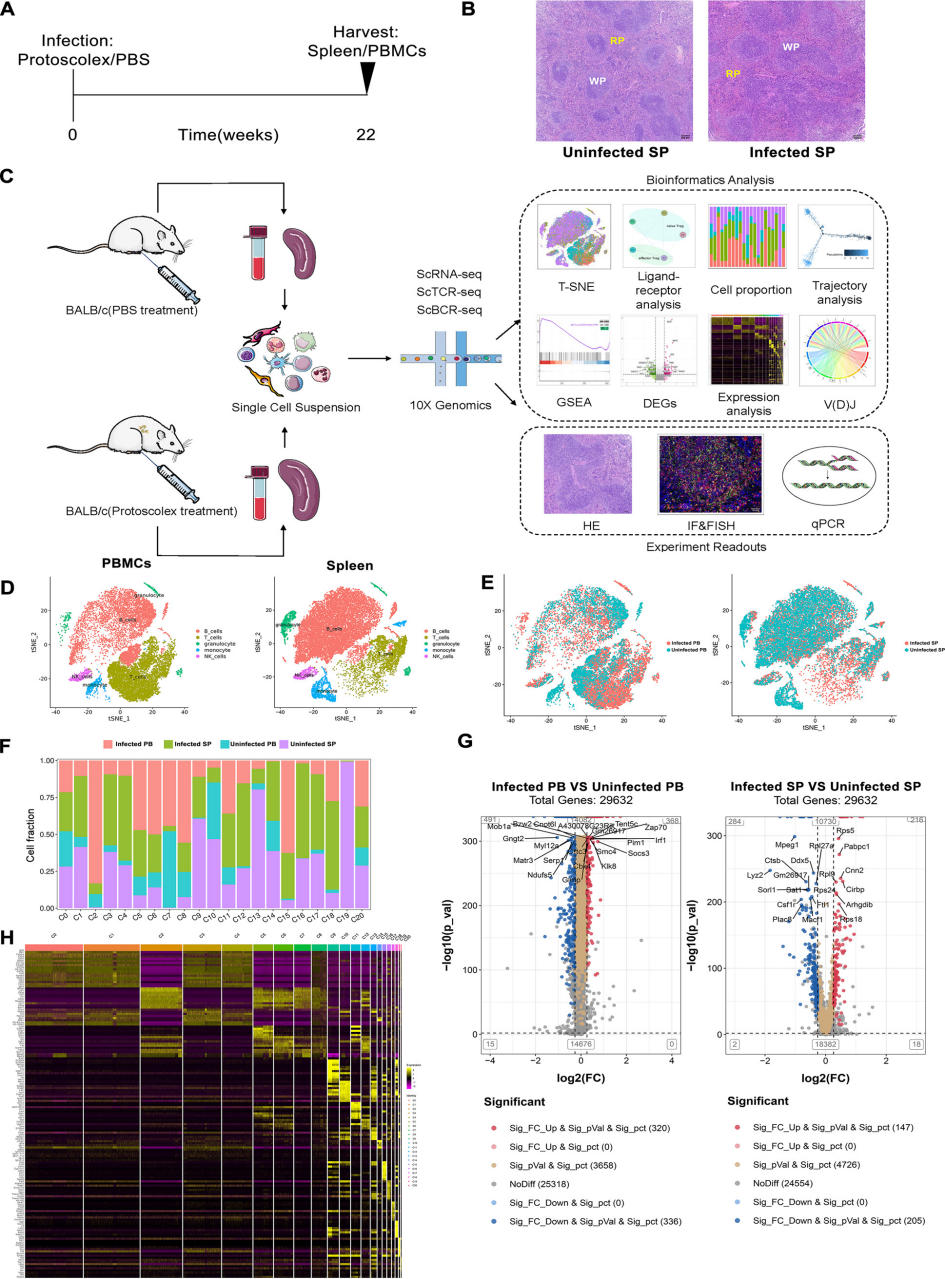
Tissue-common and tissue-specific immune system alterations. (A) Construction of the *E. granulosus* mouse model (*n* = 3 mice). (B) Hematoxylin and eosin (HE)-stained sections of spleens from uninfected and infected mice (×4 magnification, scale bar = 500 μm). (C) Research flow chart (each group is 3 mice pooled); IF, immunofluorescence; FISH, fluorescence *in situ* hybridization. (D) t-SNE plot of differential changes in immune cells in PBMCs and spleens. (E) t-SNE plot of the distribution of all immune cells from different samples. (F) Plot of cell number proportion of different clusters in four samples. Horizontal coordinates represent different clusters, and vertical coordinates represent the percentage of different sample cell numbers in each cluster. (G) Volcano plot of differential gene expression of different samples. Sig_FC_Up & Sig_pVal & Sig_pct represent genes that meet significant differential upregulation, significant differential *P* value, and percentage of cells with significantly differentially expressed genes in the infected versus uninfected groups. Sig_FC_Up & Sig_pct represent upregulation of the difference in FC value only. Sig_pVal & Sig_pct represent upregulation of the difference in *P* value only. NoDiff represents no significant difference. Sig_FC_Down & Sig_pct represent downregulation of FC value difference only. Sig_FC_ Down & Sig_pVal & Sig_pct represent genes that meet significant differential downregulation, significant differential *P* value, and percentage of cells with significantly differentially expressed genes in the infected versus uninfected groups. The number in parentheses represents the number of genes that meet this screening condition. (H) Heat map of top 10 marker gene expression in each cluster; yellow highlighting represents genes upregulated in expression, and different color bars in the upper part represent different clusters.

Well-vitalized protoscoleces were isolated from patients with confirmed CE and quantitatively injected into the abdominal cavities of mice, which were euthanized at 22 weeks of late-stage infection (Fig. 1A; Fig. S1 and S2 in the supplemental material). As the largest immune organ in mice, the spleen is located in the meridian of blood circulation, and its size and structure can reflect the body’s immune status. We observed that the spleens of uninfected mice (uninfected SP) had a clear structure, and the red pulp (RP) and white pulp (WP) were clearly distinguished. Furthermore, lymph nodules consisting of tightly arranged lymphocytes and RP containing a large number of erythrocytes were observed in hematoxylin and eosin staining of uninfected SP. However, in the spleens of infected mice (infected SP), we noted structural changes, which included irregularly enlarged WP and a relative reduction in the size of the RP (Fig. 1B). This phenomenon indicated a certain degree of change in the immune systems of mice after infection with *E. granulosus* at the late stage. Therefore, we performed scRNA-seq on mouse spleens and PBMCs to further investigate the immunological changes induced by *E. granulosus* infection. To characterize the cellular components of the immune system, we obtained 53,298 cell profiles (15,400 cells from the infected PBMCs group [infected PB], 16,765 cells from the infected SP group, 7,398 cells from the uninfected PBMCs group [uninfected PB], and 13,735 cells from the uninfected SP group; each group consists of 3 mice pooled) and 21 cell populations via scRNA-seq. The total number of genes detected in all cells was 99,442 (Fig. 1C; Fig. S3).

The raw data were quality counted using Cell Ranger analysis software. The sequenced saturation of each sample was more than 40%, with an average number of reads per cell exceeding 20,000 (Table S1). Samples with lower sequencing depth were used as a benchmark, and reads were randomly selected from samples with higher sequencing depth to homogenize the sequencing data and ultimately identify the cell expression matrix. Captured cells with low activity were filtered to further improve the quality of the analysis (Fig. S4 and Table S2). After removal of low-activity cells, the distribution of major immune cells in individual tissues was shown by principal-component analysis downscaling using *t*-distributed stochastic neighbor embedding (t-SNE) clustering analysis. We used the reference data set provided by SingleR to annotate all cells in the four samples into five different types (Fig. 1D and E). Comparisons revealed significant differences in the proportion of immune cells and gene expression levels in PBMCs and spleens between mice infected with *E. granulosus* and uninfected mice (Fig. 1F to H). We aligned the reads with the *E. granulosus* reference genome to detect the presence of *E. granulosus* transcripts in populations of five different cell types. We found that T cells, B cells, monocytes, natural killer (NK) cells and granulocytes were all affected to various degrees by *E. granulosus*-derived genes (Fig. S5). To investigate the full spectrum of immune cells in mice after *E. granulosus* infection, we subsequently reclassified these major cell types separately.

### A specific subpopulation of CD4^+^ T cells with type I interferon production potential (IFN-act T_N_) was increased in PBMCs and spleens after infection with *E. granulosus*.

**FIG 2:**
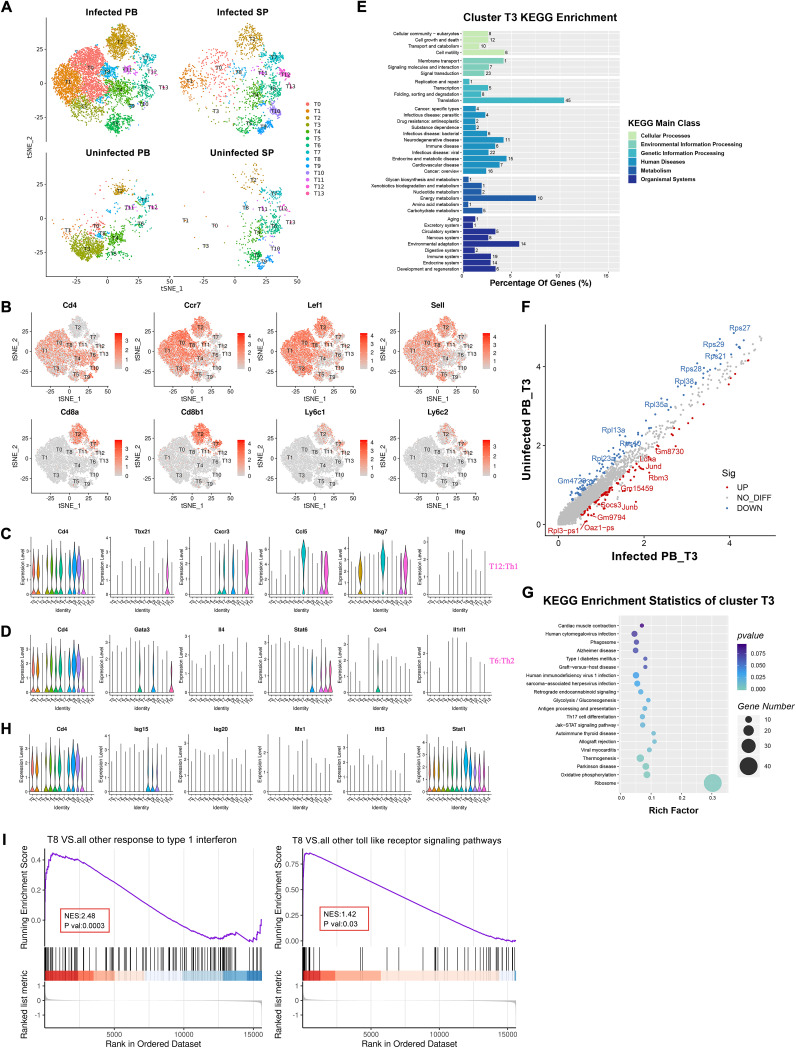
Changes in T cells occurring in PBMCs and the spleen after infection with *E. granulosus* in mice. (A) t-SNE plots of T cell subpopulation distribution in different samples. (B) t-SNE plots of T cell marker gene expression of naive CD4^+^, naive CD8^+^, and cytotoxic CD8^+^ T cells. (C and D) Violin plots of Th cell marker gene expression. (C) Th1 marker gene expression plot. (D) Th2 marker gene expression plot. (E) Cluster T3 DEGs from infected PB versus uninfected PB KEGG enrichment plot. (F) Analysis of cluster T3 DEGs in infected PB versus uninfected PB. Blue indicates genes that were downregulated in expression in infected PB compared to uninfected PB, red indicates genes that were upregulated in expression, and gray indicates genes that have no significant difference. (G) KEGG enrichment plot of cluster T3 DEGs in infected PB versus uninfected PB. Rich factor indicates the number of differentially expressed genes located in this KEGG/total number of genes located in this KEGG. (H) Maker gene expression plot in cluster T8 associated with the type I IFN response. (I) GSEA plot comparing cluster T8 to all other T cell subpopulations for genes involved in the response to type I IFN and Toll-like receptor signaling pathway. Normalized enrichment score (NES) indicates the enrichment fraction.

We screened 13,236 T cells for clustering, and the results indicated that these cells showed a differential distribution of cell types, proportions, and gene expression in different immune tissues. (Fig. 2A).

We classified T cells into CD4^+^, CD8^+^, and γδT cells (Fig. 2B; Fig. S6). Cluster T13 was increased after infection, with a low expression of *Cd4* and high expression of *Tcrg-c1*, presumably as γδT cells. Clusters T0, T1, T3, T4, and T8 were defined as naive CD4^+^ T cells due to high expression of *Cd4*, *Ccr7*, *Lef1*, and *Sell*, and, similarly, *Cd8a*, *Cd8b1*, *Ccr7*, *Lef1*, and *Sell* were highly expressed in cluster T2, which was consistent with the characteristic gene expression of naive CD8^+^ T cells. Concurrently, we observed that cluster T7 was enriched in *Cd8a*, *Cd8b1*, *Ly6c1*, and *Ly6c2*; therefore, we speculated that cluster T7 was cytotoxic CD8^+^ T cells (Fig. 2B). Violin plots demonstrate some Th cell signature genes, with cluster T12 characterized by the expression of *Tbx21* (T-bet), *Cxcr3*, *Ccl5*, and *Nkg7*, suggesting that it consists of Th1 cells. *Gata3*, *Il1rl1* (ST2), *Ccr4*, and *Pparg* were highly expressed in cluster T6, similar to the gene expression signature in Th2 cells (Fig. 2C and D) (22). Compared with uninfected PB, infected PB showed newly appeared clusters T9, T10, and γδT (T13) cells and decreased naive CD4^+^ T (T3) and Th1 (T12) cells. Furthermore, we observed that Th1 (T12) cells were slightly decreased in PBMCs, but Th2 (T6) cells were increased both in PBMCs and the spleen after infection (Fig. 2A; Table S3). In our results, the cellular immune responses of both Th1 (T12) and Th2 (T6) cells were present in *E. granulosus*-infected mice, and the percentage of Th2 (T6) cells was significantly higher in the spleens of CE mice, supporting previous findings on the transfer of Th1-type immune responses to Th2-type responses in late CE (15).

Naive CD4^+^ T (T3) cells were decreased in infected PB compared with uninfected PB. Analysis of differentially expressed genes (DEGs) in naive CD4^+^ T (T3) cells in infected PB versus uninfected PB showed enrichment of DEGs in pathways associated with endocrine and metabolic disease, immune disease, and parasite infection (Fig. 2E). Moreover, some genes were specifically highly expressed in infected PB, such as *Junb*, *Ldha*, and *Socs3* (Fig. 2F). *Junb* regulates the expression pattern of IL-4, with a bias toward Th2 directional differentiation (23). *Ldha* is associated with the HIF-1 signaling pathway, and it has been documented that knockdown of HIF-1α enhances the tumor-killing capacity of NK cells and upregulates expression levels of IFN-γ, granzyme B, and Cd107a in splenic NK cells (24, 25). *Socs3* was enriched in the JAK-STAT signaling pathway (KEGG: 4630) (Fig. 2G; Table S4). *Socs3*, via binding to JAK and cytokine receptors to inhibit STAT3, hinders JAK activation and suppresses inflammation and T cell proliferation, and overexpression of *Socs3* in T cells of transgenic mice leads to increased frequency of Th2 cells (26). Furthermore, *Socs3* and *Junb* are associated with osteoblast differentiation (Table S4). Therefore, we suggest that *Cd4*^+^*Junb*^hi^*Ldha*^hi^*Socs3*^hi^ naive CD4^+^ T cells may differentiate into Th2 cells in the future and that this subpopulation has the potential to affect bone development in *E. granulosus*-infected mice.

Cluster T8 was characterized by the expression of *Isg15*, *Isg20*, and *Ifit3*, suggesting that it may be associated with the type I IFN response (Fig. 2H). A cytokine profile shift toward Th1 and a significant reduction in lesions in patients with hepatitis C and alveolar echinococcosis treated with IFN-α has been demonstrated (27). In experimentally infected mice treated with IFN-α2a, 7 of 12 mice had significantly reduced larval damage and modulated cytokine secretion (28). To further confirm that cluster T8 may respond to type I IFN, we performed differential gene set enrichment analysis (GSEA) of cluster T8, and the results showed that DEGs were significantly associated with the type I IFN response. Furthermore, cluster T8 had a high enrichment fraction of the Toll-like receptor signaling pathway, suggesting that T8 may have the ability to produce type I IFN (Fig. 2I) (29). Therefore, we defined cluster T8 as IFN-act T_N_ cells. However, *Ifnar1* and *Ifnar2* were lowly expressed in IFN-act T_N_ (T8) cells (Fig. S7); therefore, we indicated that IFN-act T_N_ cells possibly secrete type I IFN after infection with *E. granulosus* and produce a nonpreferential immune response. The increased number of IFN-act T_N_ (T8) cells in the infected group, along with a significant enrichment of genes responding to type I IFN, suggest that we could use IFN-act T_N_ cells as a potential drug treatment target for CE in the future.

### scRNA-seq revealed differential changes in Treg subpopulations before and after infection with *E. granulosus* in mice.

**FIG 3:**
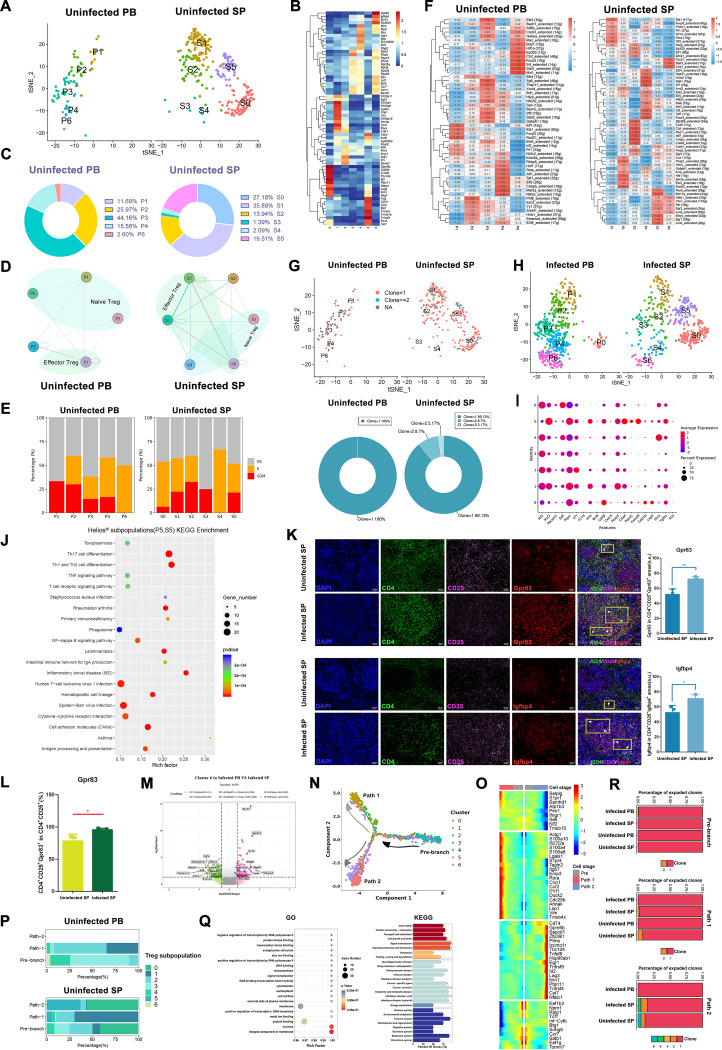
Differential changes in Treg subpopulations before and after infection with *E. granulosus* in mice. (A to G) Uninfected PB versus uninfected SP samples. (A) t-SNE plots of Treg distribution in uninfected samples. (B) Heat map of marker gene expression for each Treg subpopulation. (C) Percentage of cells in Treg subpopulations. (D) Cellular interactions of Treg subpopulations. (E) The cell cycle of Treg subpopulations. (F) Transcription factor expression profiles of Treg subpopulations. (G) Clonal expansion t-SNE and donut plots of Treg subpopulations. Red, blue, and gray represent clone = 1, clone ≥ 2, and clone = 0, respectively. (H) t-SNE plots of Treg distribution in infection samples infected PB and infected SP. (I) Treg subpopulations marker gene bubble plot. (J) Scatterplot of Helios^hi^ subpopulation (P5 and S5) KEGG enrichment analysis. The rich factor indicates the number of differentially expressed genes located in this KEGG/total number of genes located in this KEGG. (K) Immunofluorescence combined with RNA fluorescence *in situ* hybridization results. DAPI, CD4, and CD25 represent immunofluorescence stains. *Gpr83* and *Igfbp4* represent RNA probes. The white arrows indicate triple-positive cells. The statistical plot’s horizontal coordinate represents the group, and the vertical coordinate represents the mean fluorescence intensity value of the positive region (*n* = 3 views). (L) Percentage of CD4^+^CD25^+^*Gpr83*^+^ cells among CD4^+^CD25^+^ cells in infected and uninfected spleens (*n* = 3 views). (M) Volcano plot of Treg subpopulation 6 differential gene expression from infected PB and infected SP. The number in parentheses represents the number of genes that meet this screening condition. (N) Plots of differentiation trajectories of Treg subpopulations. Cells with similar statuses were clustered together, and the branch point represents a possible decision point in the cell biological process. (O) Heat map of genes specifically expressed in three different differentiation pathways. (P) Percentage of Treg subpopulations in different differentiation pathways in uninfected PB and uninfected SP samples. (Q) GO/KEGG of differentially expressed genes with a *q* value of <0.05 in the trajectory analysis. (R) Percentage of Treg cells with and without clonal expansion in different samples and at different stages of differentiation; *, *P* < 0.05; **, *P* < 0.01; ***, *P* < 0.001; ****, *P* < 0.0001.

*Foxp3* was highly expressed in both T5 and T9 populations, similar to Treg cells (Fig. S8); therefore, we reclustered 1,313 cells derived from clusters T5 and T9 to further investigate the mechanisms of negative immune regulation in mice after infection with *E. granulosus*. Likely due to the unique tissue microenvironment, there were seven subpopulations with distinct phenotypes or activation states in PBMCs and the spleen. Treg subpopulations 0 and 5 were absent in uninfected PB, and Treg subpopulation 6 was missing in uninfected SP (Fig. 3A; Table S5). Cluster 2 highly expressed *Il7r* (CD127), which we presumed was not a Treg cell but a conventional T (Tconv) cell (30); so, we defined the other cells as CD4^+^CD25^high^Foxp3^high^CD127^low^ Treg cells. Treg subpopulations 3, 4, and 6 in PBMCs (P3, P4, and P6) and subpopulations 3, 4, and 6 in the spleen (S3, S4, and S6) were *Sell*^hi^*Ccr7*^hi^*Lef1*^hi^ naive Treg cells, and Treg subpopulations P0, P1, and P5 and S0, S1, and S5 were effector Treg cells (Fig. 3B). We found a tight cellular interaction between naive Treg cells and effector Treg cells (Fig. 3C and D). High expression of *Lag3* was present in Treg subpopulations P0 and S0, named Lag3^hi^ effector Treg subpopulations; meanwhile, *Foxp3* was most highly expressed in the P1 and S1 subpopulations, which were designated Foxp3^hi^ effector Treg subpopulations. Moreover, the high expression of *Ikzf2* (Helios) in P5 and S5 subpopulations was defined as Helios^hi^ effector Treg subpopulations. The majority of P6 cells were in G_1_+S phase, and this Treg subpopulation specifically expressed the suppressor genes *Gpr83* and *Igfbp4*, which were defined as *Gpr83*^+^*Igfbp4*^+^ naive Treg cells; meanwhile, P4 and S4 highly expressed *Il2ra* (CD25) and were named CD25^hi^ naive Treg cells. P3 and S3 showed different cell cycle states. P3 was in S phase, while S3 was mostly in G_1_+G_2_/M phase (Fig. 3B to E). These Treg subpopulations showed distinct transcription factor expression profiles in uninfected PBMCs and the spleen (Fig. 3F). To gain insight into the clonal relationships between individual Treg cells, we reconstructed their T cell receptors (TCRs). We obtained full-length TCRs with α and β chains for 1,175 cells, of which 1,091 cells contained unique TCRs, and 84 cells had duplicated TCRs. Most of the amplified TCR clonotypes (*n* ≥ 2) were detected in effector subpopulations (indicating a clonal expansion), that is, the Lag3^hi^ (P0 and S0), Foxp3^hi^ (P1 and S1), and Helios^hi^ (P5 and S5) subpopulations, with few clonal expansions in subpopulations from naive Treg cells. This clonal expansion was more prominent in the Treg subpopulations of the spleen, showing some tissue specificity (Fig. 3G). However, this may also be due to the relatively low number of cells in Treg, which would also show less clonal expansion after reclustering. Together, these results define the classification of Treg subpopulations in one or two tissues and the relationship between them, enriching our understanding of Treg cells.

We noticed that the Lag3^hi^ subpopulation (P0 and S0) newly appeared in infected PB, with an increased percentage of cells from the Foxp3^hi^ subpopulation (P1 and S1), T_conv_ population (P2 and S2), cluster 3 (P3 and S3), CD25^hi^ naive Treg cells (P4 and S4), and *Gpr83*^+^*Igfbp4*^+^ naive Treg cells (P6 and S6). In the spleen, we found that *Gpr83*^+^*Igfbp4*^+^ naive Treg cells (P6 and S6) newly appeared in mice after infection, with an increased number of cells from the Lag3^hi^ subpopulation (P0 and S0), the T_conv_ population (P2 and S2), cluster 3 (P3 and S3), CD25^hi^ naive Treg cells (P4 and S4), and the Helios^hi^ subpopulation (P5 and S5) and a decreased quantity of cells from the Foxp3^hi^ subpopulation (P1 and S1). The composition of Treg subpopulations in the same tissue was not identical in healthy and infected mice, a phenomenon that suggests increased cellular heterogeneity following *E. granulosus* infection in mice (Fig. 3H). Except for cluster 3 and Helios^hi^ subpopulations (P5 and S5), other Treg subpopulations were conserved in infected PB and infected SP. Compared with uninfected PB, three different naive Treg subpopulations had an enhanced correlation in infected PB (Fig. S9). Meanwhile, GSEA results showed that the DEGs of three naive Treg subpopulations were mainly enriched in ribosomes, and the other three effector Treg subpopulations were enriched to various degrees in NK cell-mediated cytotoxicity, regulation of the actin cytoskeleton, and cytokine-cytokine receptor interaction (Fig. S10). For individual differential gene expression analysis, we found that the Helios^hi^ subpopulation (P5 and S5) was characterized by the expression of *Ctla4*, *Tgfb1*, *Icos*, *Pdcd1*, and *Tnfrsf9* (Fig. 3I), similar to the available literature findings that Treg cells with high expression of *Tnfrsf9* have higher expression levels of genes related to inhibitory functions (31). The high expression of *Tnfrsf9* (4-1BB) in the Helios^hi^ subpopulation (P5 and S5) suggests that it may be the Treg subpopulation that exerts a true negative regulation function in CE infection. Meanwhile, the Helios^hi^ subpopulation (P5 and S5) proportion was elevated in the spleens of infected mice, suggesting that *Tnfrsf9* may be another reliable marker in the clinical setting of CE. In our research, the differential gene expression analysis results using the KEGG pathway database showed that the Helios^hi^ subpopulation (P5 and S5) was associated with Th1 and Th2 cell differentiation, Th17 cell differentiation, inflammatory bowel disease, and leishmaniasis, hinting that the Helios^hi^ subpopulation (P5 and S5) may interact with Th1 or Th17 cells to suppress immune effects (Fig. 3J). For the increased number of *Gpr83*^+^*Igfbp4*^+^ naive Treg cells (P6 and S6) observed in PBMCs and spleens of infected mice, we also used immunofluorescence combined with RNA *in situ* hybridization to corroborate the specific high expression in this Treg subpopulation; the mean fluorescence intensity of *Gpr83* or *Igfbp4* and the percentage of CD4^+^CD25^+^*Gpr83*^+^ in CD4^+^CD25^+^ cells were significantly higher in the spleens of infected mice than in the spleens of uninfected mice (Fig. 3K and L) (32, 33). Differential gene expression analysis of *Gpr83*^+^*Igfbp4*^+^ naive Treg cells (P6 and S6) in infected PB and infected SP showed that *Klf2* was significantly upregulated in infected PB (Fig. 3M). *Klf2* controls naive Treg cell migration patterns by regulating homeostatic and inflammatory homing receptors, and Treg cells lacking *Klf2* do not efficiently migrate to secondary lymphoid organs (34). Therefore, we considered that in infected mice, *E. granulosus* induced a broad subpopulation of migration-competent *Gpr83*^+^*Igfbp4*^+^ naive Treg subpopulations (P6 and S6) in the spleen and peripheral blood.

The results of the trajectory analysis showed that Treg subpopulations had a total of three differentiation paths: prebranch (e.g., *Eef1b2*, *Eef1g*, and *Snhg8*), path 1 (e.g., *Actg1*, *Emp3*, and *Lsp1*), and path 2 (e.g., *Ptms*, *Gpm6b*, and *Lag3*) (Fig. 3N and O). The different Treg subpopulations merged into one process in pseudotime, naive subpopulations (mainly in the prebranch) and effector Treg subpopulations (mainly distributed in paths 1 and 2). Path 1 was mostly composed of Foxp3^hi^ (P1 and S1) and T_conv_ populations (P2 and S2), while path 2 was primarily composed of Lag3^hi^ (P0 and S0) and Helios^hi^ (P5 and S5) subpopulations (Fig. 3P). Gene Ontology (GO) enrichment of significant DEGs (*q* < 0.05) revealed that these genes were associated with the nucleus and integral membrane components, and KEGG analysis showed that they were associated with signal transduction and immune system diseases (Fig. 3Q). During the path 2 stage of Treg cell differentiation in infected mice (mainly Lag3^hi^ [P0 and S0] and Helios^hi^ [P5 and S5] subpopulations), there was a significant clonal expansion of Treg cells in the spleen (Fig. 3R). These phenomena suggest that the effector Treg subpopulations play different roles in *E. granulosus* infection, and we speculate that Lag3^hi^ (P0 and S0) and Helios^hi^ (P5 and S5) subpopulations may play more prominent roles than Foxp3^hi^ (P1 and S1) subpopulations.

### Differential gene expression analysis showed that increased amounts of Tfh2 are specifically enriched for *Cxxc5* and *Spock2* after infection.

**FIG 4:**
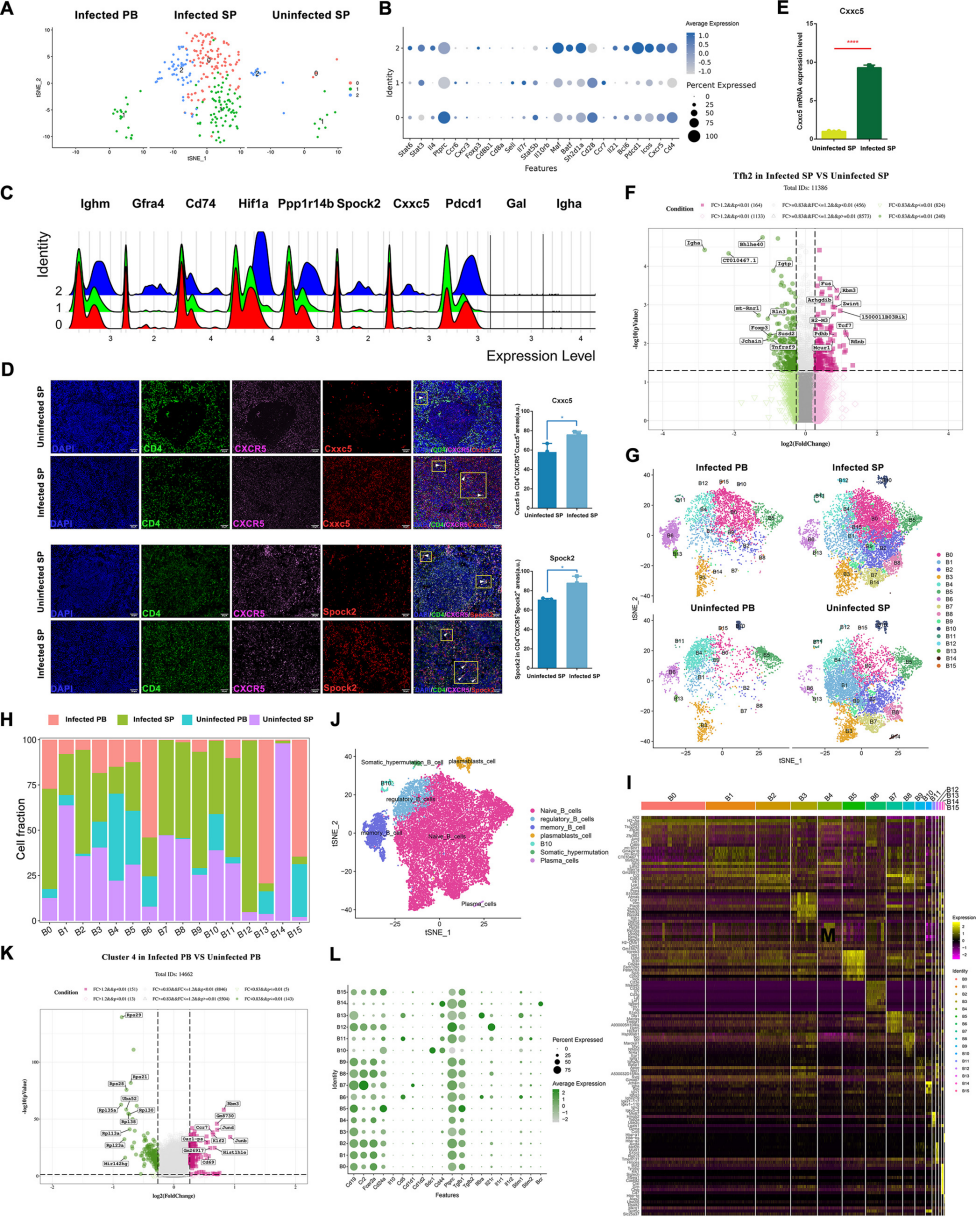
Differential analysis of Tfh and B cell subpopulations between *E. granulosus*-infected and uninfected mice. (A to F) Changes in Tfh cells. (A) t-SNE plots of Tfh cell distribution in different samples. (B) Bubble plot of Tfh cell marker gene expression distribution. (C) Tfh2 top 10 differential gene expression peak plots. (D) Immunofluorescence combined with RNA fluorescence *in situ* hybridization results. DAPI, CD4, and CXCR5 represent immunofluorescence stains. *Cxxc5* and *Spock2* represent RNA probes. The white arrows indicate triple-positive cells. The statistical plot’s horizontal coordinate represents the group, and the vertical coordinate represents the mean fluorescence intensity value of the positive region (*n* = 3 views). (E) qRT-PCR statistics of *Cxxc5* (*n* = 3). (F) Volcano plot of differential gene expression of Tfh2 cells in infected SP versus uninfected SP. The number in parentheses represents the number of genes that meet this screening condition. (G) t-SNE plots of B cell distribution in different samples. (H) Plot of the proportion of cells in different B cell subpopulations in the four samples. (I) Heat map of top 10 marker gene expression in each B cell subpopulation. (J) t-SNE plot of B cell subpopulation distribution in all samples after annotation. (K) Volcano plot of B4 differential gene expression in infected PB versus uninfected PB. (L) Bubble plot of B cell subpopulation marker gene expression distribution; *, *P* < 0.05; **, *P* < 0.01; ***, *P* < 0.001; ****, *P* < 0.0001.

*Cxcr5* and *Pdcd1* were enriched in cluster T10, hinting that T10 consists of Tfh cells (Fig. S8). To further investigate the immunomodulatory mechanisms arising in mice after infection with *E. granulosus*, we reclustered 281 cells derived from Tfh cells (*Cd4*^+^*Cxcr5*^+^*Pdcd1*^+^), resulting in the generation of three Tfh subpopulations (Tfh was not detected in uninfected PB). Compared with uninfected mice, cluster 1 was newly present in infected PB, and the numbers of clusters 0 and 2 were significantly increased in the spleen (Fig. 4A). We then annotated these cells using marker genes (35). Cluster 2 was *Cd4*^+^*Cxcr5*^+^*Icos*^+^*Il21*^+^*Il4*^+^*Bcl6*^+^*Ccr6*^−^*Cxcr3*^−^, which we defined as Tfh2, and cluster 0 was *Cd4*^+^*Cxcr5*^+^*Icos*^+^*Il21*^+^ *Ccr6*^+^*Cxcr3*^low^, which was similar to the expressed genes of Tfh17 cells (Fig. 4B). Tfh2 and Tfh17 cells could induce naive B cell differentiation more efficiently after infection, which in turn leads to differentiation of antigen-specific B cells into plasma and memory cells that produce antibodies to neutralize and clear pathogens (36).To research the immune response mechanism of Tfh subpopulations after infection with *E. granulosus*, we analyzed the top 10 highly expressed genes and found that Tfh2 specifically overexpressed *Cxxc5* and *Spock2* genes (Fig. 4C). Furthermore, we confirmed this sequencing result using immunofluorescence combined with RNA *in situ* hybridization; the mean fluorescence intensity of *Cxxc5* or *Spock2* was significantly higher in CD4^+^CXCR5^+^ cells from the spleens of infected mice than in the spleens of uninfected mice; the mRNA expression of *Cxxc5* also showed the same trend (Fig. 4D and E). Analysis of Tfh2 differentially expressed genes in infected SP versus uninfected SP revealed the upregulated expression of *Fus* in infected SP (Fig. 4F). The lack of *Fus* in mice could lead to defective B lymphocyte development and activation (37), implying that Tfh2 may play a role in promoting B cell activation, proliferation, and differentiation in *E. granulosus* infection.

It has been shown that the decrease in germinal center cells is partly due to the loss of the Tfh cell phenotype, and these cells may differentiate into memory cells in the future (38). In our study, cluster 1 lowly expressed *Cxcr5*, *Pdcd1*, and *Bcl6* genes and reexpressed *Ccr7* and *Sell* (CD62L), which is most similar to memory Tfh cells. Therefore, we believe that these memory Tfh cells could rapidly and efficiently promote the formation of germinal centers and antibodies during contact with *E. granulosus*-associated antigens (38).

### scRNA-seq indicated that mice infected with *E. granulosus* produce four Breg subpopulations and a population of TGF-β^+^B220^+^CD23^+^CD21^+^CD24^low^CD138^−^ B cells with suppressive potential.

As different types of immunoglobulins were produced at different stages of infection, we performed an in-depth analysis of 25,780 B cells to study the immune response produced by B cells after infection with *E. granulosus*. B cell subpopulations showed different states in different tissue samples (Fig. 4G). Compared with uninfected PB, clusters B12 and B14 newly appeared in infected PB, with a decrease in the proportion of clusters B4, B5, and B10 and an increase in the remaining clusters. For the spleen, a different mapping emerged after infection, with a decrease in the amounts of clusters B1, B3, B4, B5, and B14 and an increase in the remaining clusters (Fig. 4H). We annotated these cells according to differentially expressed genes in each cluster: naive B cells (*Cd19*^+^*Ighd*^+^), dividing B cells (*Mki67*^+^*H2afz*^+^*Cdk1*^+^), memory B cells (*Cd19*^+^*Cd27*^+^*Ighd*^+^), and somatic hypermutation B cells (*Aicda*^+^) (Fig. 4I and J) (39). We then noticed that cluster B12 was somatic hypermutation B cells, which had an elevated proportion in infected SP and was newly present in infected PB; thus, the role of somatic hypermutation B cells in infection with *E. granulosus* could be to promote antibody diversity and affinity maturation (40). Cluster B11 was dividing B cells, and clusters B6 and B13 were memory B cells; these cell types were growing in both infected PB and infected SP, suggesting that humoral immunity was also in an active phase at this time.

The numbers of cluster B4 were decreased in infected PB, and analysis of their differentially expressed genes revealed that cluster B4 was enriched in *Cd69* after infection (Fig. 4K). Modulation of the innate immune system with monoclonal antibodies to host CD69 provides a novel means to antagonize tumor growth and metastasis (41). CD69 is crucial to the immunosuppressive function of Treg cells by promoting IL-10 production (42). In *E. granulosus* infection, high expression of *Cd69* by cluster B4 implied that this cluster may play an immune regulatory role in suppressing inflammation. The expressed pattern of B4 was *Tgfb1*^+^ (TGF-β), *Ptprc*^+^ (B220), *Fcer2a*^+^ (CD23), *Cr2*^+^ (CD21), *Cd24a*^low^ (CD24), and *Sdc1*^−^ (CD138) (Fig. 4L). This led us to turn our attention to regulatory B cells, which negatively regulate the immune response. After analysis of differentially expressed genes in B cells, we identified clusters B6, B10, B11, and B14 as Breg subpopulations (43). Cluster B10 was plasmablast cells (*Sdc1*^+^*Cd44*^hi^), clusters B6 and B11 were B10 cells (B cells that produce IL-10; *Cd5*^+^*Cd1d1*^+^*Cd1d2*^+^), and cluster B14 was plasma cells (*Sdc1*^+^*Ptprc*^+^) (Fig. 4L). The numbers of clusters B10 and B14 in PBMCs and the spleen showed the opposite trend, which also verified that Breg cells were not genealogically specific but a subpopulation of cells responsive to inflammation, and any B cell can differentiate into a Breg cell; furthermore, after inflammation subsides, Breg cells can further differentiate into other cells, such as plasma cells (43). We also counted regulatory B cell immunoglobulin subtypes (Fig. S11). Briefly, mice were induced with four Breg subpopulations after infection with *E. granulosus*, and their cell proportions showed different distribution trends. Plasma cells were increased in infected PB, and B10 (B cells that produce IL-10) were increased in infected PB and infected SP, suggesting that these two Breg subpopulations may play an important role in the induction of *E. granulosus* immune escape, further reminding us that Breg cells are also involved in the dynamic balance of immune cells in long-term chronic inflammation caused by *E. granulosus*.

### Single-cell V(D)J sequencing of T and B lymphocytes showed specific gene fragment expression downregulation of multiple TCR β-chain V-J pair combinations in mice infected with *E. granulosus*.

**FIG 5:**
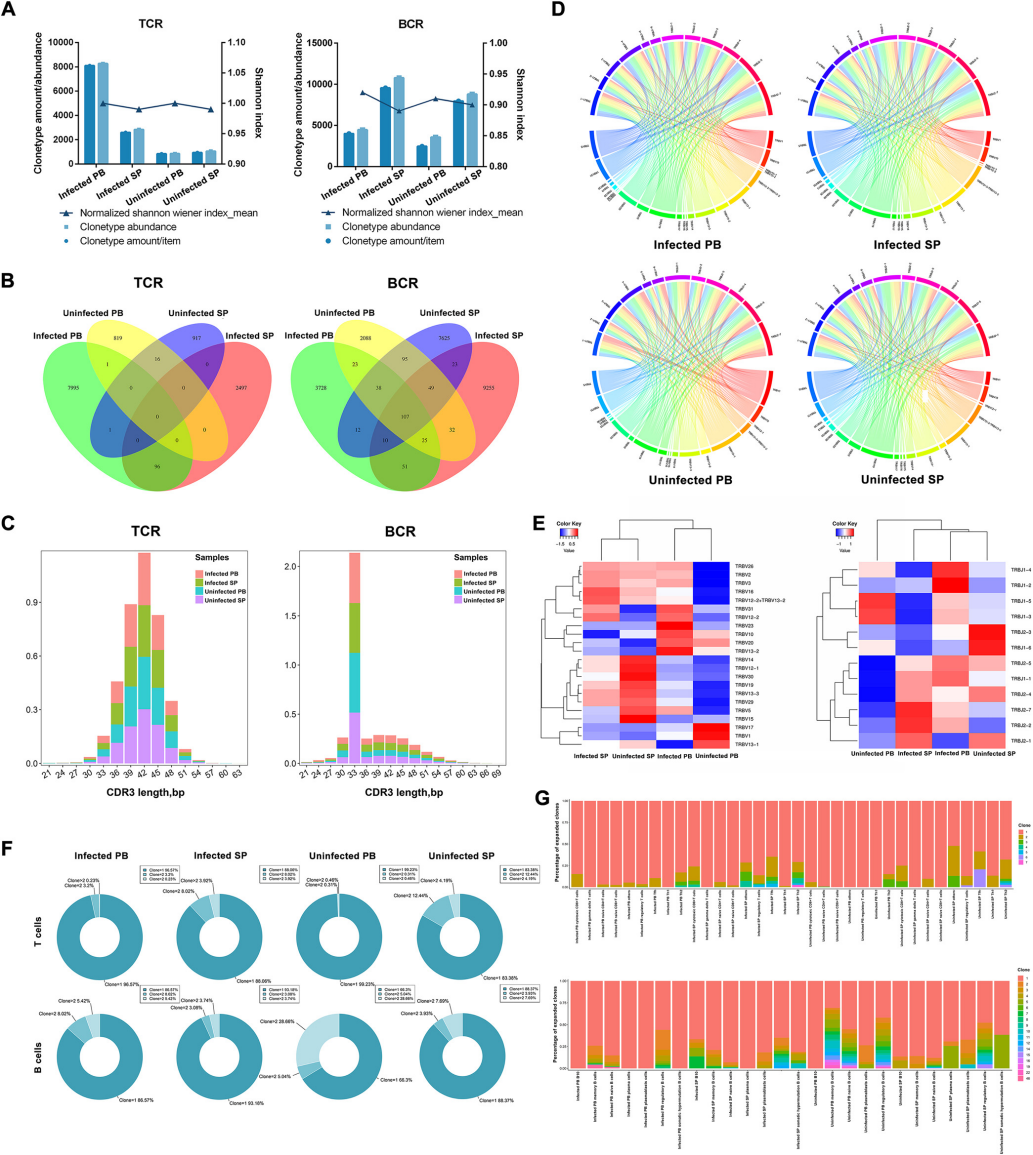
Differential changes in TCRs/BCRs in mice after infection with *E. granulosus* (each group is 3 mice pooled). (A) Changes in TCR/BCR clonotype amount, abundance, and normalized Shannon index for different samples. (B) Venn diagrams of the number of TCR/BCR common (overlapping) and unique (nonoverlapping) clonotype amounts between different samples. (C) CDR3 polypeptide length distribution of TCRs/BCRs. (D) Circos plots of TRB V-J paired frequency in the TCR. The outermost arc indicates V and J genes. Each color block represents one gene; wider color blocks represent higher gene frequencies. The connecting arcs between inner color blocks indicate V-J pairs. (E) Heat map of TRB V gene (left) and TRB J gene (right). (F) TCR/BCR clone donut plots in different samples. (G) Percentage of TCR/BCR clones of cell subpopulations in different samples.

To deeply explore the recognition, processing, presentation, and signaling of *E. granulosus*-associated antigens by T and B cells *in vivo* after infection with *E. granulosus* in mice, single-cell immunome repertoire (IR) sequencing was performed. We noticed that clonotype amount, clonotype abundance, and clonal diversity showed differential alterations between PBMCs and the spleen, infection and health, and TCRs and B cell receptors (BCRs). Clonotype amount and abundance of TCRs/BCRs in PBMCs and the spleen increased differentially after infection, with a larger increase in TCR clonotype count and abundance in infected PB (Fig. 5A). We also observed that TCRs had few common clonotypes before and after infection compared to BCRs, which indicated a lower TCR similarity and overlap rate between infected and uninfected mice (Fig. 5B). This was also consistent with our sustained immunological studies of *E. granulosus* infection, in which T cells play a greater role in the fight against *E. granulosus*.

T cells are one of the main performers of the body’s immune functions. TCRs are molecules on the surface of T cells that recognize antigens, and the molecule that best represents characteristics of the T cell response is TCR high-variable region complementary determining region 3 (CDR3). CDR3 is encoded by a portion of variable (V), all diversity (D), and joining (J) and the linkage region between V-D and D-J and therefore has the highest degree of diversity and is the focus of attention for IR. Statistics on the distribution of CDR3 region peptide lengths in different samples of TCRs/BCRs revealed that the peptide lengths of TCR CDR3 were mainly distributed in the interval of 36 to 48 bp and were uniformly distributed in different samples, while for BCRs, the peptide lengths of the CDR3 region in each sample were mainly concentrated at 33 bp (Fig. 5C). Vβ and Jβ gene fragments are major components of the CDR3 area; therefore, the β-chain V-J pairs from different samples were counted (Fig. 5D). We observed that 73 V-J pairs were specifically expressed in infected PB, and 36 V-J pairs were specifically expressed in infected SP; 1 V-J pair was specifically expressed in uninfected PB, and 6 V-J pairs were specifically expressed in uninfected SP. However, these V-J pairs occurred less frequently (frequency range of 1 to 33). Meanwhile, we found some β-chain V-J pairs specifically expressed in infected PB and infected SP, which accounted for a greater proportion of the top 10 downregulated expression levels of the TRBV (TRBV17, TRBV15, TRBV30, TRBV12-1, TRBV14, and TRBV29) and TRBJ (TRBJ1-5, TRBJ1-3, TRBJ1-6, and TRBJ1-4) genes after infection (Fig. 5E).

Combined analysis of IR and transcriptomic data revealed that the clonotypes of several T and B cell subpopulations were altered after infection in mice (Tables S6 and S7), especially in PBMCs that exhibited clonal expansion of some T cells, mainly including naive CD8^+^ T cells and Treg cells. As classical Th1/Th2 immune cells after *E. granulosus* infection, we also observed alterations in their clonotypes in different samples, with a clonal expansion of Th2 cells in PBMCs after infection (Fig. S12). For B cells, we found that mice showed similar clonal expansion of B10 (B cells that produce IL-10) and memory B cells in the spleen after infection (Fig. 5F and G). This result suggests that targeting and correcting Th2, Treg, and Breg cells, which favor parasite-induced immune escape, during late *E. granulosus* infection is an important focus of our attention. A point worth raising is that this clonal expansion phenomenon may be specific to the mouse due to the small number of cells, but these results still give us very important clues.

### scRNA-seq indicated a homeostatic immunoregulatory state of myeloid cells in *E. granulosus*-infected mice.

**FIG 6:**
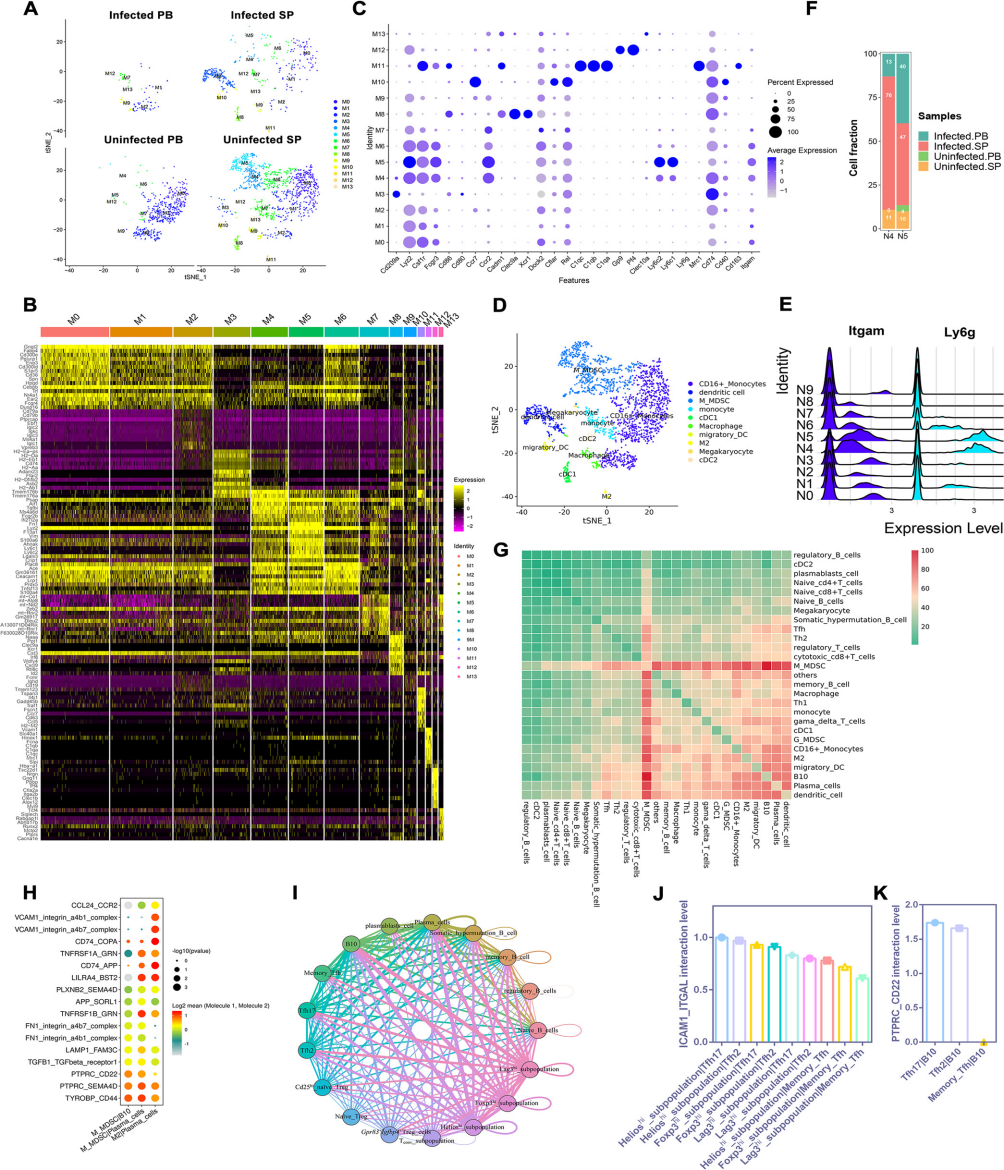
Analysis of myeloid cell subpopulations and interaction network. (A to D) Differential changes in monocyte subpopulations. (A) t-SNE plots of monocyte subpopulation distribution in different samples. (B) Heat map of top 10 marker gene expression in each monocyte lineage subpopulation. (C) Bubble plot of monocyte marker gene expression distribution. (D) t-SNE plot of monocyte subpopulation distribution in all samples after annotation. (E and F) Differential analysis of granulocyte subpopulations. (E) G-MDSC marker gene expression peak plots. (F) Percentage of granulocyte cell subpopulations N4 and N5 in different samples. (G to K) Cellular communication. (G) Heat map of interactions between different cell types. Heat maps were plotted as the number of protein interactions between different cell subpopulations (*P* ≤ 0.05). (H) M-MDSCs-B10 cells, M-MDSCs-plasma cells, and M2 cells-plasma cells had significantly differentially expressed top 10 interacting proteins. (I) Interaction network diagram of Treg, Tfh, and B cell subpopulations. Different-colored circles represent different cell types; the thickness of the line represents the interaction index, high (thick) or low (thin). (J) Comparison of ICAM1-ITGAL protein interaction pairs in effector Treg and Tfh cell subpopulations. (K) Comparison of PTPRC-CD22 protein interaction pairs in B10 and Tfh cell subpopulations.

To more fully demonstrate the immune system changes that occur after infection of mice with *E. granulosus*, we analyzed 2,505 monocytes and 2,166 granulocytes from different tissues and observed that most monocytes showed a decrease in cell proportions following infection (Fig. 6A). Analysis of differentially upregulated genes in each cluster showed that clusters M0, M1, and M2 expressed similar gene patterns, as did clusters M4, M5, and M6 (Fig. 6B). We defined clusters by categories. Clusters M0, M1, and M2 (*Fcgr3*^+^*Csf1r*^+^*Lyz2*^+^) were CD16^+^ monocytes, clusters M4, M5, and M6 (*Itgam*^+^*Ly6c*^+^) were monocytic myeloid-derived suppressor cells (M-MDSCs), cluster M3 (*Cd209a^+^Cd74*^+^*Cd40*^+^*Cd80*^+^*Cd86*^+^) was dendritic cells (DCs), cluster M11 (*Mrc1^+^C1qa*^+^*C1qb*^+^*C1qc*^+^) was M2-like macrophages, cluster M7 (*Lyz2*^+^*Csf1r*^+^*Ccr2*^+^) was monocytes, cluster M9 was macrophages (*Lyz2*^+^*Dock2*^+^), cluster M10 (*Cflar*^+^*Ccr7^+^Rel*^+^) was migratory DCs (44), cluster M12 was megakaryocytes (*Gp9*^+^ *Pf4*^+^), cluster M8 (*Clec9a*^+^ *Xcr1*^+^*Cadm1*^+^) was type I classical DCs (cDC1s), and cluster M13 (*Clec10a*^+^) was type II classical DCs (cDC2s) (Fig. 6C and D) (45, 46). Among the reclustering granulocytes, we noticed high expression of *Itgam* with *Ly6g* in clusters N4 and N5, which was consistent with characteristic markers of granulocytic myeloid-derived suppressor cells (G-MDSCs) (Fig. 6E).

Our cellular annotation results showed a significant decrease in CD16^+^ monocytes after infection. Compared with uninfected SP, infected SP showed increased DCs, decreased M2-like macrophages, and decreased cDC1s. Similarly, cDC2s were newly emerged in infected PB compared with uninfected PB. The decrease in CD16^+^ monocytes and cDC1s may make organisms less capable of antigen presentation. After *E. granulosus* infection, there was an increase in G-MDSCs and a decrease in M-MDSCs (Fig. 6A and F), which suggests that MDSCs play a nonnegligible immunosuppressive role in the middle and late stages of *E. granulosus* infection; meanwhile, this effect was different for M-MDSCs and G-MDSCs. M2-like macrophages induce anti-inflammatory responses and promote immune escape of parasites in the host. The decrease in M2-like macrophages suggests that the M1/M2 balance is disrupted in the fight against *E. granulosus* infection and that the specific mechanisms of these immune cells need to be further investigated in the future.

In brief, we saw that immune regulation of myeloid cells in mice infected with *E. granulosus* was extremely complex; whether proinflammatory or anti-inflammatory, pathogen cleared or immune escape induced, it was a relative equilibrium of the organism rather than a polarization of the state. This “abnormal balance” leads to chronic infection and persistent *E. granulosus* disease.

### Ligand-receptor analysis disclosed interactions between the Helios^hi^ Treg subpopulation-Tfh17-B10 cell regulatory axis and M-MDSCs with multiple immune cells.

We conducted a ligand-receptor-based cellular interaction analysis to explore the direct and indirect mechanisms of immune cell interrelations in mice after infection with *E. granulosus*. The results revealed that M-MDSCs interacted with a variety of cells, and among the highest interaction index was M-MDSCs-B10 cells, followed by M-MDSCs-plasma cells (Fig. 6G). Further analysis of the interacting proteins showed that the ligand-receptor pair with the highest interaction index was TYROBP-CD44, followed by PTPRC-SEMA4D and PTPRC-CD22 (Fig. 6H). When associated with different receptors, DAP12 (TYROBP) has been shown to enhance and attenuate leukocyte activation (47). Knockdown of semaphorin 4d (Sema4D) in a human head and neck squamous cell carcinoma (HNSCC) cell line resulted in a loss of MDSC function, as shown by a decrease in the production of the immunosuppressive cytokines arginase-1, TGF-β, and IL-10 by MDSCs. Meanwhile, human HNSCC-associated Sema4d induces expansion of MDSCs (48). CD22 is a B cell-restricted surface protein that regulates BCR signaling in mature B cells. As an inhibitory coreceptor, CD22 inhibits BCR-induced Ca^2+^ signaling, while CD45 is a prominent CD22 binding partner (49). CD22 is an attractive molecule for B cell-targeted therapy. Anti-CD22 therapeutic antibodies have been developed in different forms, and an antibody called epratuzumab has been clinically studied in B cell malignancies, such as non-Hodgkin’s lymphoma and systemic lupus erythematosus (50). These data show that M-MDSCs play a nonnegligible immunosuppressive role in *E. granulosus* infection.

During the analysis of M-MDSCs-plasma cells, it was found that the pair index LILRA4-BST2 was high, which regulated activation of immune cells. Nevertheless, this receptor-ligand pair was not the highest in M-MDSCs-plasma cells, and in-depth research revealed that its reciprocal index in M2 (M2-like macrophages)-plasma cells was highest (Fig. 6H; Fig. S13). It has been shown that BST2 is an IFN-inducible cytokine induced by IFN-α, and IFN-α secretion is controlled by the interaction between LILRA4 (ILT7) and BST2 (51). Interestingly, the type I IFN-related genes *Ifnar1*, *Ifnar2*, and *Irf9* were highly enriched in plasma cells (Fig. S14). This indicated that induction of type I IFN production has a regulatory effect on plasma cells after infection with *E. granulosus* in mice, and M2-like macrophages play a crucial role in such regulation. Additionally, we reiterate the possible effect of type I IFN in an animal model of *E. granulosus* infection.

Since both Treg and Tfh cells expressed *Pdcd1* and *Ctla4*, and there is a close regulatory role between Tfh and B cells, we explored the relationship between Treg, Tfh, and B cells using ligand-receptor interaction analysis. Between Treg and Tfh subpopulations, *Gpr83*^+^*Igfbp4*^+^ naive Treg cells (P6 and S6) had the highest index of interactions with Tfh2 cells, and the Helios^hi^ (P5 and S5) subpopulation had the highest index of interactions with Tfh17 cells (higher than *Gpr83*^+^*Igfbp4*^+^naive Treg cells [P6 and S6]-Tfh2 cells), while Tfh17 and B10 cells had a close reciprocal relationship (Fig. 6I; Table S8). Further analysis of the Helios^hi^ (P5 and S5) subpopulation-Tfh17 cells and Tfh17 cells-B10 cells with significant differences in the reciprocal proteins showed that their strongest reciprocal indexes were intercellular adhesion molecule 1-integrin alpha L (ICAM1-ITGAL) and PTPRC-CD22, respectively (Fig. 6J and K). ICAM1 and ITGAL, previously referred to as leukocyte function associated molecule 1, promote the recruitment of inflammatory cells to the feto-maternal interface and trigger abortion (52). ITGAL (CD11a) is essential for inflammation and immune responses and regulates the adhesion and costimulatory interactions between CD4^+^ T cells and other cells. Studies of systemic sclerosis have revealed that overexpression of CD11a in CD4^+^ T cells may mediate the immune abnormalities and fibrotic processes of the disease and show a possible relationship between this molecule and B cells (53). Therefore, we hypothesized that a Helios^hi^ subpopulation-Tfh17-B10 cell regulatory axis may be formed between Treg, Tfh, and B cells via ICAM1-ITGAL and PTPRC-CD22 to trigger immune evasion responses to *E. granulosus* in mice.

## DISCUSSION

In this study, we present a panoramic view of immune cell changes following host infection with *E. granulosus* and focus attention on immune cell changes that favor host parasitism, such as Treg cells, Breg cells, and MDSCs. In contrast to conventional studies of Treg cell changes in *E. granulosus* infections, our results showed that seven Treg subpopulations with different phenotypes or states were induced in PBMCs and spleens of infected mice, and these subpopulations had different gene expression and differentiation characteristics. The *Gpr83*^+^*Igfbp4*^+^ naive Treg subpopulation that negatively regulates the immune system and *Cxxc5*^+^*Spock2*^+^ Tfh2 cells, which promote B cell-mediated humoral immunity, were first identified during *E. granulosus* infection. In scTCR-seq, some β-chain gene fragments were downregulated, and there was clonal expansion of Th2 and Treg cells. These results further enriched the study of the mechanism of bidirectional immune regulation after *E. granulosus* infection. Meanwhile, our study provides mechanistic insights and an important reference for further understanding of the immune landscape during the late stage of *E. granulosus* infection and a reference value for studying the potential disease mechanisms of CE and finding possible therapeutic targets.

Our preliminary analysis of the data revealed that both PBMCs and spleens were enriched with a large number of immune cells; however, heterogeneity was still observed. Therefore, we classified T lymphocytes, B lymphocytes, myeloid cells, and granulocytes for processing analysis in different samples. The immune effects produced by type I IFN in parasites are currently not fully understood, and some studies have reported that IFN-α/β may have host-protective or deleterious effects in malaria, depending on the stage of infection and *Plasmodium* (54). Type I IFN promotes DC activation and increases the expression of cell surface major histocompatibility complex (MHC) class II molecules with costimulatory factors CD80 and CD86, thereby enhancing their antigen-presenting ability; furthermore, type I IFN can act directly on CD4^+^ and CD8^+^ T cells and B cells to increase their activation levels (29). It has also been shown that type I IFN causes humoral immune dysfunction and impairs the antigen-specific response (55). However, some studies have shown that IFN-α in combination with albendazole may help treat human and animal CE (56). In this study, we noticed increased numbers of specific CD4^+^ T cell populations in infected PB and infected SP that had upregulated expression of type I IFN response genes after infection with *E. granulosus* and high expression of *Ifnar1*, *Ifnar2*, and *Irf9* in B cell subpopulation B14 (i.e., plasma cells [*Sdc1*^+^*Ptprc*^+^]) (Fig. S14), indicating that *Sdc1*^+^*Ptprc*^+^ Breg cells could respond adequately to type I IFN. Therefore, we propose that type I IFN may play an important role in *E. granulosus* infection, and its effect pathway has a higher correlation with *Sdc1*^+^*Ptprc*^+^ Breg cells, but its specific immunomodulatory mechanism must be further investigated.

Predominantly Th1-type cells and cytokines are involved in the early stages of *E. granulosus* infection, with a shift to a Th2-type response in the late stages of infection (17–19). We know that Th2 cells inhibit the development of Th1 cells by inhibiting the function of IFN-γ, mainly through the secretion of IL-4 (15, 57). In this study, we identified a population of naive CD4^+^ T (T3) cells with high expression of *Junb*, *Ldha*, and *Socs3* in infected PB that have the potential to differentiate to Th2 cells in the future. *Junb* and *Socs3* are associated with osteoclast differentiation and show similar results to bone loss in mice after schistosome infection (58–61). However, bone loss has not been reported in *E. granulosus* infection, and there has been only one case of bone involvement after Echinococcus multilocularis infection (62). Therefore, whether *E. granulosus* infection causes bone loss and whether the bone loss is associated with Th2 cells is a question we need to investigate in depth in the future.

Treg cells act as “immune brakes” *in vivo*, causing tumor immune escape due to the expression of suppressive genes such as *Pdcd1* and *Ctla4* (63). Treg cells perform a similar immune efficacy in *E. granulosus* infections (64). With the development of single-cell transcriptome sequencing technology, Treg cells have been increasingly studied. Luo et al. classified Treg cells into nine subpopulations in human graft-versus-host disease (65). Our results for *E. granulosus* infection are similar in that Treg cells consist of seven different subpopulations, mainly naive Treg cells and effector Treg cells, and each of them belongs to a different stage of differentiation and has tissue specificity. Relatively high expression of Treg cell-related regulatory genes was in the Helios^hi^ Treg subpopulation (P5 and S5), implying that the Helios^hi^ subpopulation (P5 and S5) may truly exert negative immunomodulatory functions. We proposed that the immunosuppressive effect after *E. granulosus* infection was achieved through the Helios^hi^ Treg subpopulation-Tfh17-B10 cell regulatory axis. Meanwhile, we observed that Treg subpopulation 6 was naive Treg cells, increased after infection, and specifically expressed *Gpr83* and *Igfbp4*. According to relevant studies, the acquisition of suppressive functions by Treg cells is associated with the expression of *Gpr83* and related suppressor molecules, such as *Foxp3*. Furthermore, *Gpr83* is involved in the peripheral induction of CD4^+^Foxp3^+^ Treg cells during the active immune response, and T cells with retroviral transduction of *Gpr83* acquire *Foxp3* expression *in vivo* and exhibit immunosuppressive functions under inflammatory conditions (33, 66). However, it has also been shown that *Gpr83* plays a nonessential role in Treg cell development and function (67). Thus, it remains to be further investigated whether the expression of *Foxp3* was induced by *Gpr83* or whether *Gpr83* has a facilitative or nonessential role in Treg cell development and function. *Igfbp4* has a potent growth and invasion inhibitory function, which is associated with transcriptomic alterations leading to deregulation of multiple signaling pathways (68). During a study of human mesenchymal stem cells (MSCs), it was found that Igfbp4 in MSC culture supernatants inhibited Treg cell proliferation (32). In our research, the high expression of *Igfbp4* may likewise suggest an immunosuppressive function performed by Treg subpopulation 6. Meanwhile, this population also highly expressed *Rgs10*, which activates M2-like macrophages by enhancing the IL-4-induced alternative activation gene expression profile and attenuating macrophage phagocytic activity (69). Therefore, *Gpr83*, *Igfbp4*, and *Rgs10* may be important elements in the inhibitory function of Treg cells.

It has been shown that if *Cxxc5* produces a heterozygous variant, it leads to decreased levels of antibody production in the body, progressive loss of B cells, and recurrent lung and gastrointestinal infections; moreover, *Cxxc5* does not play a role in early B cell production but may affect their proliferation and survival (70). *Spock2* is a gene encoding a complex secreted proteoglycan, and its high expression in lung adenocarcinoma results in elevated proportions of tumor-infiltrating CD8^+^ T cells, activated memory CD4^+^ T cells, Treg cells, M1-like macrophages, activated myeloid dendritic cells, and neutrophils, associated with a good prognosis (71). In our study, we observed that both genes were specifically highly expressed in Tfh2 cells; therefore, we concluded that *Cxxc5* and *Spock2* may be two key regulators of Tfh2 cells in promoting humoral and cellular immunity during *E. granulosus* infection.

Mice have been shown to exhibit enhanced antitumor activity in the absence of *Cd69*, and in other diseases, the lack of *Cd69* has been associated with exacerbated forms of collagen-induced arthritis (72, 73). Furthermore, *Cd69* inhibits Th17 cell-mediated inflammatory responses (74). In an experimental model of contact dermatitis, blocking or deleting *Cd69* increased tissue damage and recruitment of Th1 and Th17 lymphocytes (75). Therefore, we noticed that TGF-β^+^B220^+^CD23^+^CD21^+^CD24^low^CD138^−^ subpopulation B4 may have a certain immunosuppressive function in *E. granulosus* infection; however, this must be corroborated by further experiments.

MDSCs are mainly comprised of M-MDSCs and G-MDSCs. M-MDSCs exert their suppressive effects by inhibiting NK cell function, inducing Treg cell expansion, and activating STAT1, which upregulates inducible nitric oxide synthase (iNOS) expression to suppress T cell production (76, 77). G-MDSCs are an important subpopulation of circulating neutrophils whose inhibitory mechanisms are primarily to suppress CD8^+^ T cells through the production of reactive oxygen species. It has been demonstrated that γδT cells promote the accumulation and expansion of G-MDSCs in patients with colon cancer (78). We typed MDSCs to explore the interactions with other immune cells and noted an increase in γδT cells in parallel with G-MDSCs. Meanwhile, analysis of ligand-receptor interactions showed that C5AR1-RPS19 was more frequent in G-MDSCs-gdT top 10 interacting proteins. C5AR1-RPS19 upregulates immunosuppressive signaling and has also been reported to induce the production of immunosuppressive cytokines (including TGF-β) via MDSCs in tumor-draining lymph nodes, resulting in a biased T cell response toward the Th2 phenotype (79). An increase in G-MDSCs after infection with *E. granulosus* indicated that M-MDSCs and G-MDSCs may have different roles in promoting the immune escape of *E. granulosus*. These phenomena also bring new concerns and research directions. Furthermore, this study echoes previous MDSC research work by our group (80). The M1/M2 pattern is similar to the Th1/Th2 response profile in Th cells (81), M1-like macrophages are key effector cells for the clearance of pathogens, viral infections, and cancer cells, and M2-like macrophages are thought to have roles in parasite inhibition, the promotion of tissue remodeling, and tumorigenesis development. As macrophages have been intensively studied in recent years, their nomenclature and classification are gradually changing; for example, M2-like macrophages can be subdivided into M2a, M2b, and M2c, which play different immunomodulatory roles, respectively. Our study showed a decrease in M2-like macrophages, and their implied immune status still needs to be further explored in the future (82, 83). It has been shown that cDC2s activate naive CD4^+^ T cells through MHC class II antigen presentation as well as costimulation, while a decrease in the number of Th17 cells was observed in a model with impaired cDC2 development and function (84). Our current study demonstrated a complex regulatory network among myeloid cells in the host following *E. granulosus* infection, with increases or decreases in both pro- and anti-inflammatory classes of cells; therefore, further refinement of the role played by each cell type in the fight against *E. granulosus* is a direction we should continue to focus on in the future. The limitation of this study is the lack of extensive experimental validation due to the difficulty in obtaining specimens, but the results of the analysis guide us in the direction of continued exploration and in-depth research in the future.

In conclusion, we used scRNA-seq to construct a single-cell transcriptional landscape in mice infected with *E. granulosus*. The unbiased bioinformatics analysis identified a distinct type of *Gpr83*^+^*Igfbp4*^+^ naive Treg cell and *Cxxc5*^+^*Spock2*^+^ Tfh2 cell. We also investigated the development of Treg subpopulations and the potential intercellular interactions of mouse immune cells. In short, our data provide insights into immune cell generation in mice infected with *E. granulosus*, which might lead to the development of clinical applications.

## MATERIALS AND METHODS

### Ethics statement.

All animal experiments in this study were approved by the Animal Ethics Committee of Ningxia Medical University Medical Ethical Committee (permit number 2019-121) and conducted in strict accordance with the Guide for the Care and Use of Laboratory Animals of the Ministry of Science and Technology of the People’s Republic of China. Human liver cyst samples were obtained with informed consent from patients with a confirmed diagnosis of *E. granulosus* at the Affiliated Hospital of Ningxia Medical University. This study was conducted in accordance with the tenets of the Declaration of Helsinki. Informed consent was obtained from all human subjects, and approval from the ethics committee for involvement of human subjects was granted.

### Animals.

Wild-type 6- to 8-week-old female BALB/c mice were purchased from the Beijing Weitonglihua Laboratory Technology, Co., Ltd. (qualification certificate: SCXK Beijing 2016-0006). Mice were housed at the Experimental Animal Center of Ningxia Medical University and raised under specific pathogen-free conditions with random access to food and water. Mice were housed individually in ventilated cages and randomly assigned to experimental and control groups of five mice each.

### Cystic echinococcosis model.

Cysts were surgically removed and opened to expose the ascospores, which were washed three times with 0.01 M PBS (Servicebio) to remove impurities before being aspirated with a syringe. The cyst fluid was allowed to stand for 30 min at 4°C to obtain precipitation of the protoscolex, and the concentration was adjusted to 20,000 cells/mL using PBS. Protoscolex motility characteristics and structural integrity were observed under the microscope. Then, mice in the experimental group were injected intraperitoneally with 2,000 protoscoleces per mouse, and mice in the control group were injected with an equal volume of PBS. After 22 weeks, mice were euthanized, and their spleens and peripheral blood were immediately isolated.

### Tissue dissociation and preparation of single-cell suspensions.

Mouse peripheral blood was placed in a disposable blood collection tube containing EDTA-K2 anticoagulant, and 1 mL of PBS was added to mix the blood thoroughly. Subsequently, 2 mL of lymphocyte isolation fluid (1.077 g/mL; Sigma) was added to a clean sterile 15-mL centrifuge tube and equilibrated at room temperature for 30 min; afterward, the centrifuge tube was tilted at a 45° angle, and 2 mL of PBS-diluted blood was slowly injected with the tip of a pipette onto the upper layer of the lymphocyte isolation fluid. We then centrifuged the suspension at 700 × *g* for 20 min at room temperature, with a boost acceleration of 9 × *g* and a deceleration-acceleration of 1× *g*. After centrifugation, the PBMC layer was aspirated, and the erythrocytes were lysed for 5 min using 0.1 M erythrocyte lysis solution (Miltenyi Biotec). The cell precipitation was resuspended in PBS containing 0.04% bovine serum albumin (BSA; Solarbio, Beijing).

Ten microliters of the PBMC suspension was taken and mixed with equal parts acridine orange and propidium iodide (RE010212, Countstar); then, 20 μL of the mixture was pipetted onto a cell counting plate, which was inserted into the Countess II automated cell counter (Countstar automatic fluorescence cell analyzer, Shanghai Ruiyu Biotechnology, Co., Ltd.) for determination of cell count and viability. When cell viability reached 85% or more, the cell concentration was adjusted to 700 to 1,200 cells/μL to build a sequencing library. If cell viability did not reach 85%, dead cell removal procedures were performed to increase the percentage of live cells, based on the manufacturer’s instructions (130-090-101, Miltenyi Biotec).

Concurrently, we obtained single-cell suspensions of the spleen by rapid grinding of the isolated mouse spleens on ice, filtration on a 70-μm filter, centrifugation at 350 × *g* and 4°C for 5 min, and removal of the supernatant. We then incubated the erythrocyte lysis solution with the cell suspensions at 4°C for 5 min and centrifuged the cell suspensions at 350 × *g* for 5 min at 4°C. We added RPMI 1640 medium (Thermo Fisher, 11875101) containing 0.04% BSA to resuspend the cell pellets. Cell quality control was performed as described above.

### Chromium 10x Genomics library and sequencing.

Cells from the spleens and PBMCs of infected (3 mice pooled) and uninfected (3 mice pooled) mice were washed once in PBS with 0.04% BSA and loaded onto the 10x Genomics Chromium to capture single cells for scRNA-seq following the manufacturer’s instructions for the 10x Genomics Chromium single-cell 5′ kit (V2.0). In brief, gel beads containing barcode information were combined with a mixture of cells and enzymes and then encapsulated by droplets of oil surfactant in a microfluidic “double-cross” system to form gel beads in emulsions (GEMs), which flowed into a reservoir and were collected. The gel beads were lysed to release the barcode sequence, cDNA fragments were reverse transcribed, and the sample was labeled. After breaking the gel beads and oil droplets, cDNA was broken into fragments of about 200 to 300 bp using enzymes, and sequencing connectors and primers were added. Finally, PCR amplification was performed to obtain the DNA library. Libraries were sequenced on an Illumina NovaSeq 6000 (LC-Bio Technologies, Co. Ltd., HangZhou, China) sequencing system using a 150-bp paired-end multiplexing run.

### Data quantification, multisample data merging, and quantitative homogenization.

Cell Ranger software (https://support.10xgenomics.com/single-cell-gene-expression/software/pipelines/latest/what-is-cell-ranger) was used to perform data quality statistics on raw data and to match the reference genome. Cell Ranger matches reads to the reference genome and combines it with the species genome annotation information, and the reads matched to the genome were classified into exons, introns, and intergenic regions (reads with at least 50% base pairing to exons, introns, and intergenic regions). When reads matched to one exon and one or several other nonexons simultaneously, the reads were preferentially classified as exon reads. If the reads matched the exons of a transcript and both were in the same direction, the transcriptome reads were considered. Transcriptome reads were unimapped if they matched to only one gene, and only unimapped reads were used for unique molecular identifier (UMI) counting. The UMI corresponding to each gene under each 10x barcode was deduplicated to obtain a unique UMI, and the number of unique UMIs indicated cellular gene expression.

This experiment involved a total of four groups (samples corresponded to multiple libraries); therefore, integration of multiplex data and homogenization of the data volume were performed before analysis. Briefly, samples with lower sequencing depths were used as a benchmark, and reads were randomly selected from samples with higher sequencing depths until all samples had the same amount of sequencing per cell on average, obtaining uniform UMI abundance information for all genes in all cells.

### Classification of single-cell subpopulations.

After removing low-quality cells, expression was homogenized using the LogNormalize method of the Seurat software “Normalization” function, and reduction of the variables was performed by the principal-component analysis (PCA) dimensionality reduction technique. Homogenized expression values were used for PCA. Furthermore, the top 10 principal components were selected from the PCA results for subsequent clustering and isolated cell population analysis, for which the following were performed: (i) the *k*-nearest neighbor (KNN) clustering relationship graph based on Euclidean distance was constructed with Seurat software using significant principal components, (ii) the weight values of intercellular distances were optimized using Jaccard similarity, and (iii) clusters were identified by a clustering algorithm optimized based on the shared nearest neighbor (SNN) module; that is, primarily KNNs were calculated and SNN graphs were constructed, then the modularization function was optimized to identify clusters. Based on the results of cell subpopulation classification, the single-cell subpopulation classification results were further visualized using the *t*-distributed stochastic neighbor embedding (t-SNE) nonlinear dimensionality reduction method.

### Marker gene analysis and cell type identification.

Seurat analysis of differentially expressed genes (DEGs) in different clusters was performed by the bimod likelihood ratio statistical test to screen for genes with upregulated expression in different clusters. The screening conditions for upregulated genes were (i) genes expressed in more than 10% of cells in the target or control subpopulation, (ii) a *P* value of ≤0.01, and (iii) gene expression ploidy log (fold change [FC]) of ≥0.26, that is, gene upregulation ploidy of ≥2^0.26^.

After identifying marker genes for each cluster classification, we identified the true cell type of each classification by marker genes, calculated the correlation between the gene expression profile of each cluster and reference data set, and obtained the cell identification category corresponding to each cluster. We also counted cell types identified by each cluster and calculated their percentage. For the difficult-to-identify clusters that we selected to manually identify again based on specific marker genes derived from the literature or cell marker database (CellMarker2.0 [http://xteam.xbio.top/CellMarker/]).

### GO and KEGG pathway enrichment analysis for DEGs.

Genes with a *q* value of <0.05 in the three Treg cell differentiation stages were mapped to each term in the GO database (http://www.geneontology.org/); differently enriched GO terms were calculated by hypergeometric testing. We subjected upregulated DEGs screened by cluster to differential gene enrichment analysis. Pathway significant enrichment analysis was performed using the KEGG pathway as a unit and applying hypergeometric testing to identify pathways that were significantly enriched in DEGs compared to the whole genomic background (www.genome.jp/kegg). A *P* value of ≤0.05 was considered to be statistically significant. The calculated *P* value was corrected by false discovery rate (FDR), and a *P* value of ≤0.05 was considered to be statistically significant.

### Ligand-receptor-based analysis of cellular interactions.

In the CellPhoneDB software library (https://www.cellphonedb.org/), we used cell expression matrices and cell types to obtain the average expression and significance relationships of ligand-receptor complexes. Single-gene proteins and protein complexes were treated separately in the CellPhoneDB. First, for single-gene proteins, we (i) removed duplicate IDs and kept only the first one, (ii) removed genes without reciprocal annotations, and (iii) removed genes that were expressed as 0 in all cells. Second, for protein complex reciprocal relationship processing, we (i) did not consider the reciprocal relationship of the complex if a subunit gene in the complex was not in the expression matrix, (ii) obtained the expression of each subunit protein gene in the complex and calculated the average expression value of the complex, and (iii) removed duplicate genes.

Based on the known cell expression matrix, the cluster gene expression matrix was obtained; the gene expression in the cluster was the average expression of the gene in all cells in the cluster. The possible cluster relationships could be inferred from gene interactions, and expression levels of interactions were calculated based on the prereceived cluster relationships. Finally, a two-dimensional matrix based on protein and cluster interactions was obtained. The random interaction matrix was obtained by randomly assigning clusters to which the cells belonged for iteration (default 1,000) and judging the number of times the expression value was greater than the observed value in all random matrices; meanwhile, the ratio of the number of iterations to the significant *P* value was used as the significant *P* value. Finally, a two-dimensional matrix containing the significant *P* value was obtained. Combining the expression and *P* value matrices eventually yielded a combined matrix, that is, the average expression matrix that demonstrated significant enrichment.

### T and B lymphocyte immune repertoire V(D)J sequencing.

After preparation of cell suspensions (each group consists of 3 mice pooled), gel beads and oil droplets were added to different lanes of a Chromium Chip A (8 lanes in total) to form a “water-in-oil” GEM via a microfluidic “double-cross” crossover system. After cell lysis, the gel beads were autolysed to release a large number of primer sequences and mixed with the cell lysate; the reaction mixture (containing reverse transcription reagents and poly[dT] reverse transcription primers) and the RNA with poly(A) were reverse transcribed to produce cDNA with 10x barcode and UMI information. The full-length cDNA was divided into two parts, and one part was subjected to nested PCR using a universal primer linked with a 5′ splice and a sequential nested primer for the T cell receptor/B cell receptor (TCR/BCR) constant region. This process, together with the sequencing splice P5, achieved enrichment of the TCR/BCR region. After amplification and enrichment of the TCR/BCR region, enzymatic fragmentation was completed, and the final sequence could cover the entire V(D)J fragment cDNA sequence by controlling the interruption of different lengths. Furthermore, the V(D)J library containing P5 and P7 junctions was constructed by PCR through end repair, addition of A, and splice ligation of read2 sequencing primers. Another copy of the amplified cDNA was segmented and screened for suitable fragments, and a 5′ expression profile library containing P5 and P7 junctions was constructed by PCR through end repair, addition of A, and splice ligation of read2 sequencing primers.

The software used for data standard analysis was Cell Ranger, officially provided by 10x Genomics, to perform bioinformatic analysis of the downstream data and construct immunome libraries, including UMI filtering, barcode screening, contig assembly, annotation and screening, consensus splicing, and visualization of the analysis results. The constructed immunome libraries were characterized, including CDR3 profiling, V/J gene profiling, and V-J paired profiling, using VDJ Tools software, and the corresponding plots were drawn.

### Immunofluorescence and RNA fluorescence *in situ* hybridization.

After preparation and digestion of paraffin-embedded sections before hybridization, we added the probe hybridization solution at a concentration of 1 μM and incubated the sections in a humidified chamber to hybridize overnight at 42°C. Then, we washed and blocked the sections and incubated the antibodies as follows. For the CD4 primary antibody, a PBS solution containing BSA (Servicebio, G5001) and a dilution of the primary antibody (CD4, 1:100; CD25, 1:100 [Servicebio, GB11612]; CXCR5, 1:200 [ABclonal, A8950]) were added and incubated at 4°C overnight. Subsequently, the samples were washed with PBS three times for 5 min each at room temperature. After washing, the sections were incubated for 50 min with the second antibody at room temperature. Then, samples were washed with PBS three times for 5 min each at room temperature. Samples were blocked by the addition of BSA and incubated at room temperature for 30 min. Sections were incubated with DAPI (Servicebio, G1012) for 8 min in the dark, and antifluorescence quenching sealer was added (Servicebio, G1401) dropwise to seal the tablet after rinsing.

Microscopic examination and photography were performed. Image acquisition was accomplished with a scanner (Panoramic slice scanner, PANNORAMIC DESK/MIDI/250/1000), and, for interpretation of the microscopic examination, the nuclei stained by DAPI were blue under UV excitation, and positive expression was fluorescence labeled by corresponding luciferin. 6-Carboxyfluorescein (FAM; 488) was green light, Cy3 was red light, and Cy5 was pink light. Three fields of view were selected for each group, and the mean fluorescence intensity was calculated using Fiji ImageJ software. Positive cell counts were calculated using Halo v3.0.311.314.

The attached probe information is as follows: *GPr83* 5′-GCACTGAGGACAGAAGAAAGAGAAGCAGGACAGGAGGAAC-3′, Cy3 labeled; *Igfbp4* 5′-CAACAACCGCAGCCTGGCTCCCGCACCAACTCC-3′ Cy3 labeled; *Cxxc5* 5′-GGGGAAAGCGCCCAGATAGGGGAAGTCGG-3′ Cy3 labeled; *Spock2* 5′-CCTTGTGGCGACTGCACTTCACCTTCTGGC-3′ Cy3 labeled.

### Hematoxylin and eosin staining.

After preparation of mouse spleen slices, samples were stained with hematoxylin staining solution (Servicebio, G1005-1) for 3 to 5 min, washed with tap water, and differentiated with differentiation solution (Servicebio, G1005-3). Subsequently, the slices were washed with tap water and returned to blue with blue return solution (Servicebio, G1005-4). Next, slices were dehydrated in 85 and 95% gradient alcohol for 5 min each and stained in eosin staining solution (Servicebio, G1005-2) for 5 min. Last, slices were placed for 5 min each into anhydrous ethanol I, anhydrous ethanol II, anhydrous ethanol III, xylene I, and xylene II transparent; then, the slices were sealed with neutral gum. Image acquisition and analysis used Panoramic slice scanner and Fiji ImageJ software, respectively.

### Quantitative real-time PCR (qRT-PCR).

TRIzol reagent was used to extract total RNA from single-cell suspensions of erythrocytes removed from mouse spleens. The isolated RNA was transcribed into cDNA by reverse transcriptase (Thermo scientific, K1622). Then, the quantification of target and reference genes was performed using SYBR green (DBI Bioscience, DBI-2043) and StepOnePlus real-time PCR instruments. Target gene expression was normalized to the expression of *Gapdh* (Sangon Biotech, B662304). The following primer sequences were used: *Cxxc5* forward, TCAGGCAAGAAGAAGCGGAAA; *Cxxc5* reverse, ACGGAAGCATCACCTTCTCCA; *Gapdh* forward, GGTTGTCTCCTGCGACTTCA; *Gapdh* reverse, TGGTCCAGGGTTTCTTACTCC.

### Statistical analysis.

Data are presented as the mean ± standard error of the mean (SEM) by GraphPad Prism 6 software (GraphPad, La Jolla, CA, USA) for statistical analysis. Differences between the two groups were analyzed by unpaired *t* tests. The results were considered statistically significant at a *P* value of <0.05. Images were created with Adobe Photoshop CS3.

### Data availability.

scRNA-seq, scTCR-seq, and scBCR-seq data are available on the Gene Expression Omnibus repository with accession numbers GSE216347 and GSE225312.
