# Supplementary material for: Single-Cell RNA Sequencing Reveals Unique Alterations in the Immune Panorama and Treg Subpopulations in Mice during the Late Stages of Echinococcus granulosus Infection
Source: Infect Immun. 2023 Apr 11;91(5):e00029-23. doi: 10.1128/iai.00029-23 (PMC10187122; doi:10.1128/iai.00029-23)
Supplement: Supplemental file 1 — Fig. S1 to S14 and Tables S1 to S8. Download iai.00029-23-s0001.docx, DOCX file, 5.1 MB [file iai.00029-23-s0001.docx]

Supplementary Material


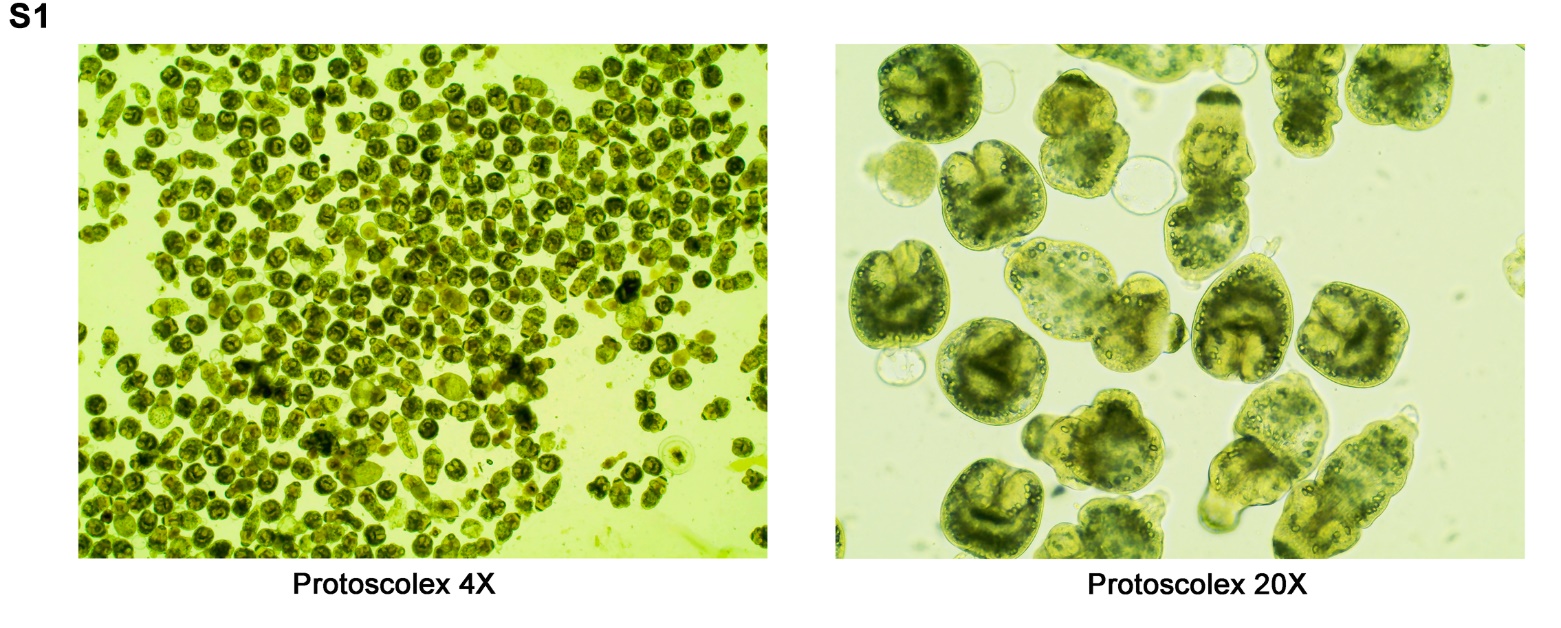


**FIG S1.** **Structure of** **protoscolex.** Protoscolex structure at 4× and 20× magnification.


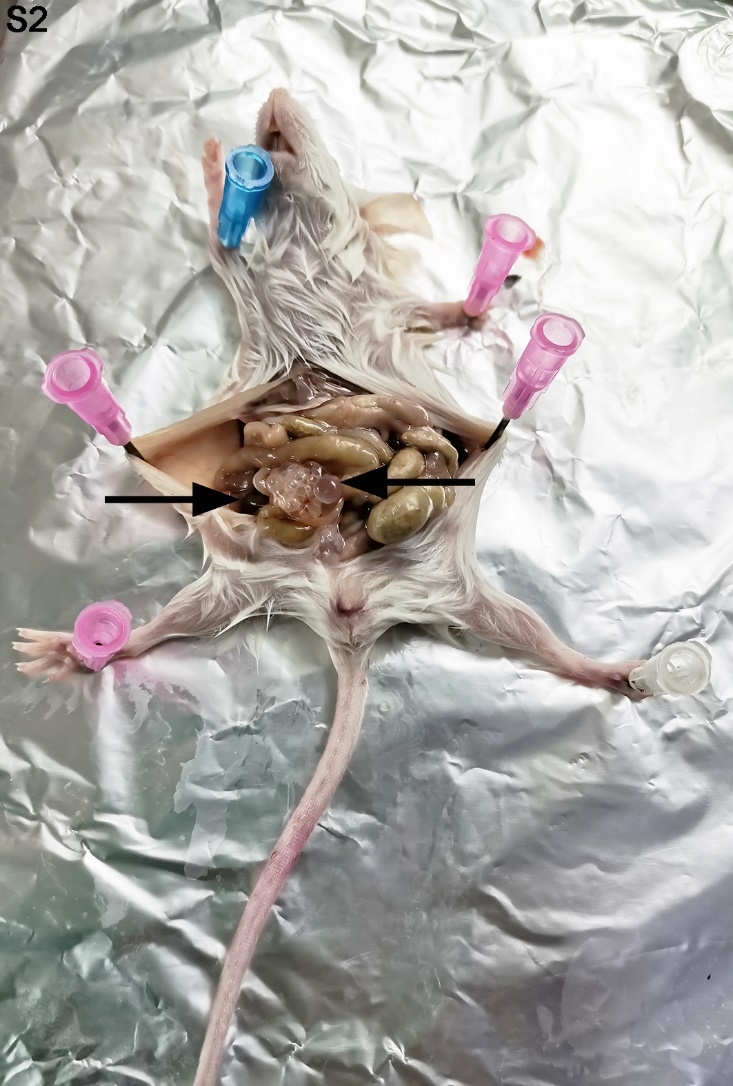


**FIG S2.** ***E. granulosus*-infected model in mice.** Anatomical view of a mouse model of secondary protoscolex infection. The black arrows point to circular semi-permeable vesicles in the lower abdomen, which are cysts.


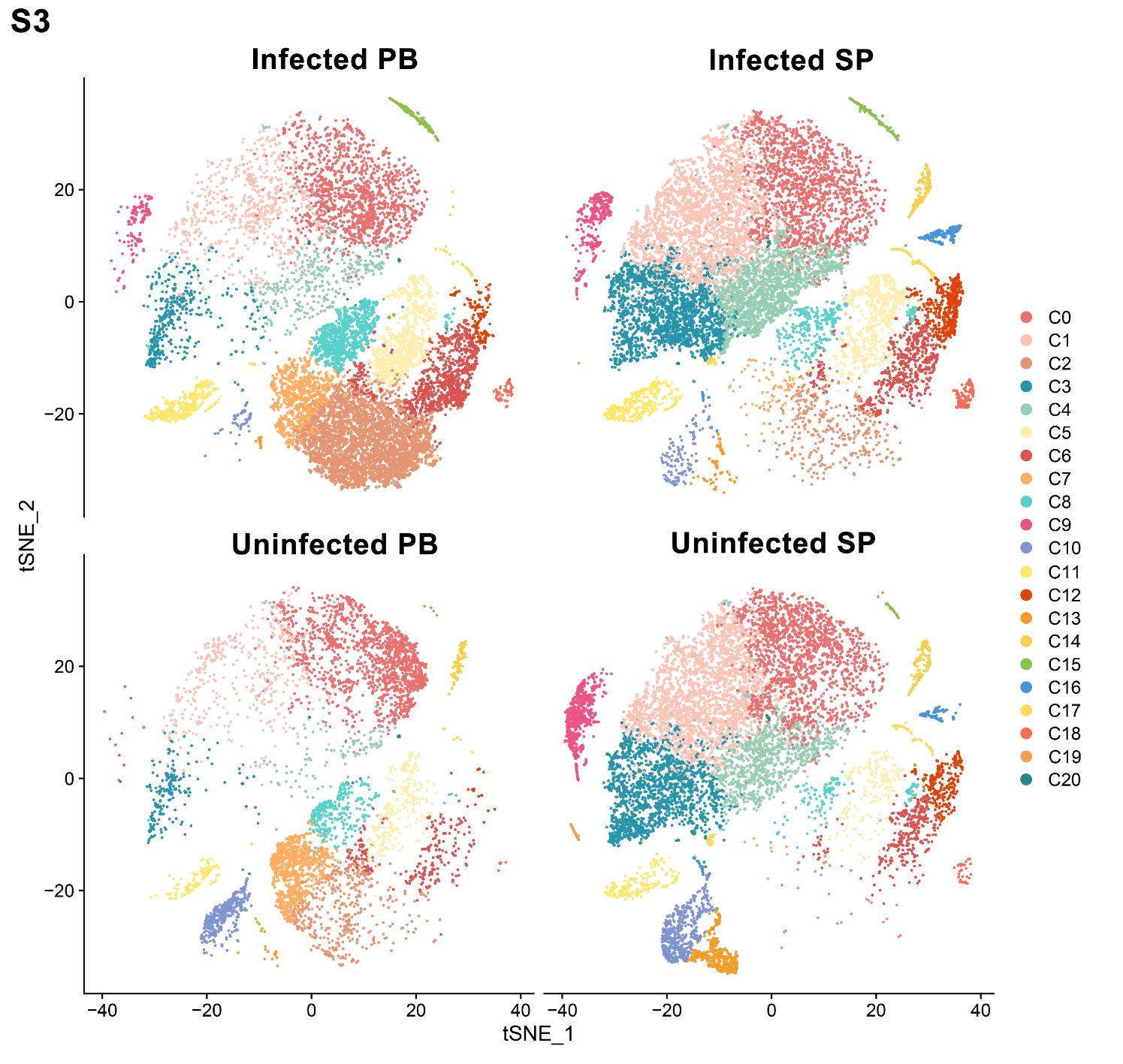


**FIG S3.** **T-SNE distribution map.** T-SNE distribution of different clusters in different samples.


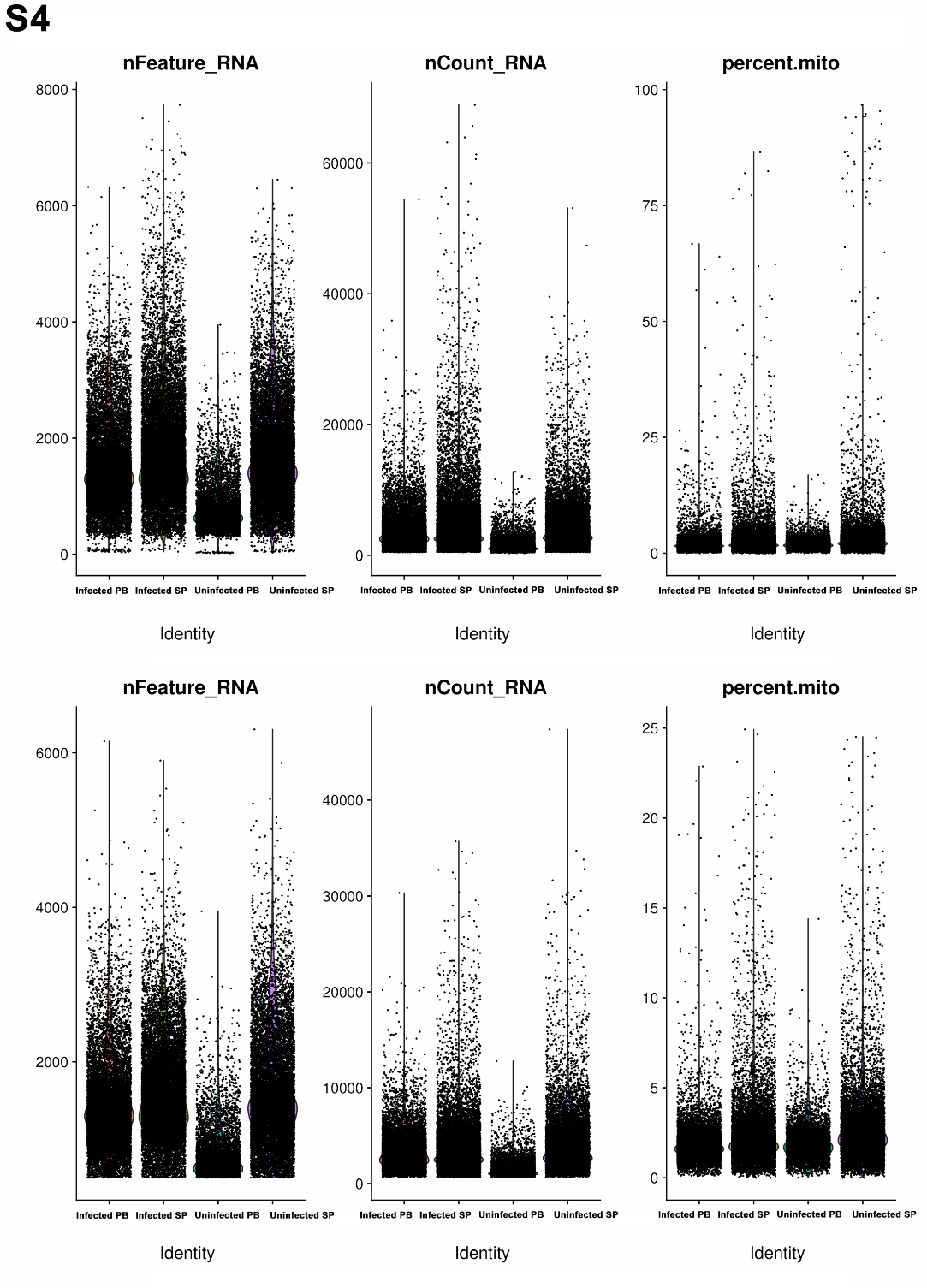


**FIG S4.** **Comparison of before and after removal of low-quality cells.** The distribution of basic information of individual sample cells before (above) and after (below) cell filtering. The left panel of each set of images shows the gene number distribution detected in individual cells for each sample (Y-axis); the middle panel shows the distribution of the total number of UMIs detected in individual cells for each sample (Y-axis); and the right panel shows the distribution of mitochondrial gene percentage expression in individual cells for each sample (Y-axis).


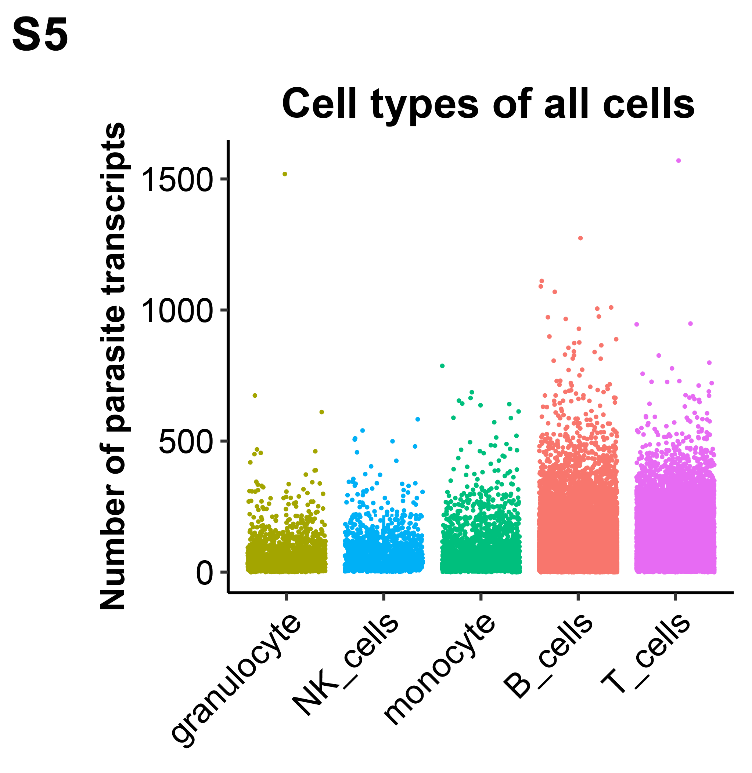


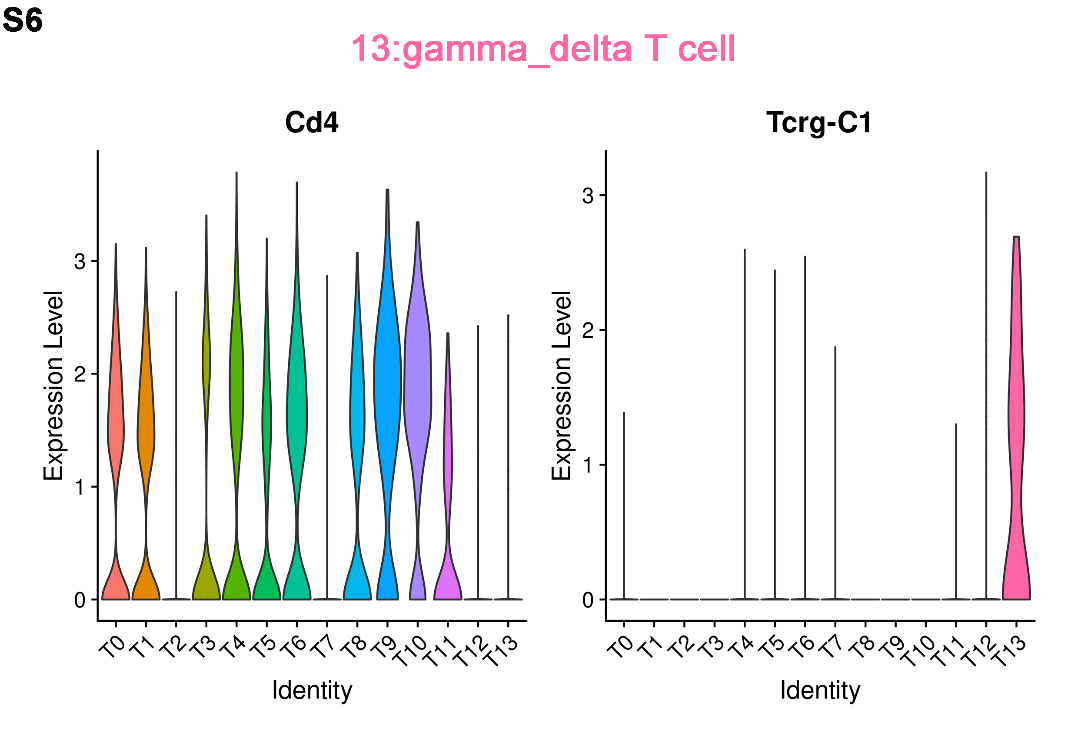
**FIG S5.** **Comparison of *E. granulosus*-derived genes with immune cell genes.** Each dot represents one cell, and different colors represent different types of immune cells.

**FIG S6. Violin plots of gamma delta T cells maker gene expression.** The title of each subplot is the marker gene name; the X-axes represent T cell subpopulations and the Y-axes represent the expression level of T cell subpopulations corresponding to the marker gene.


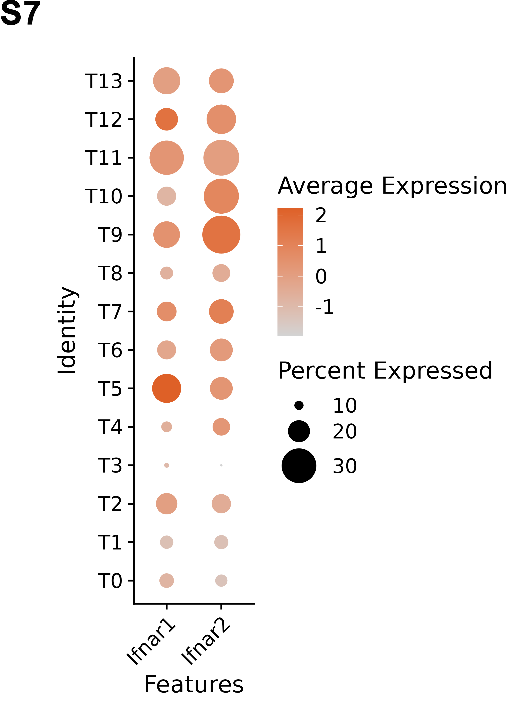


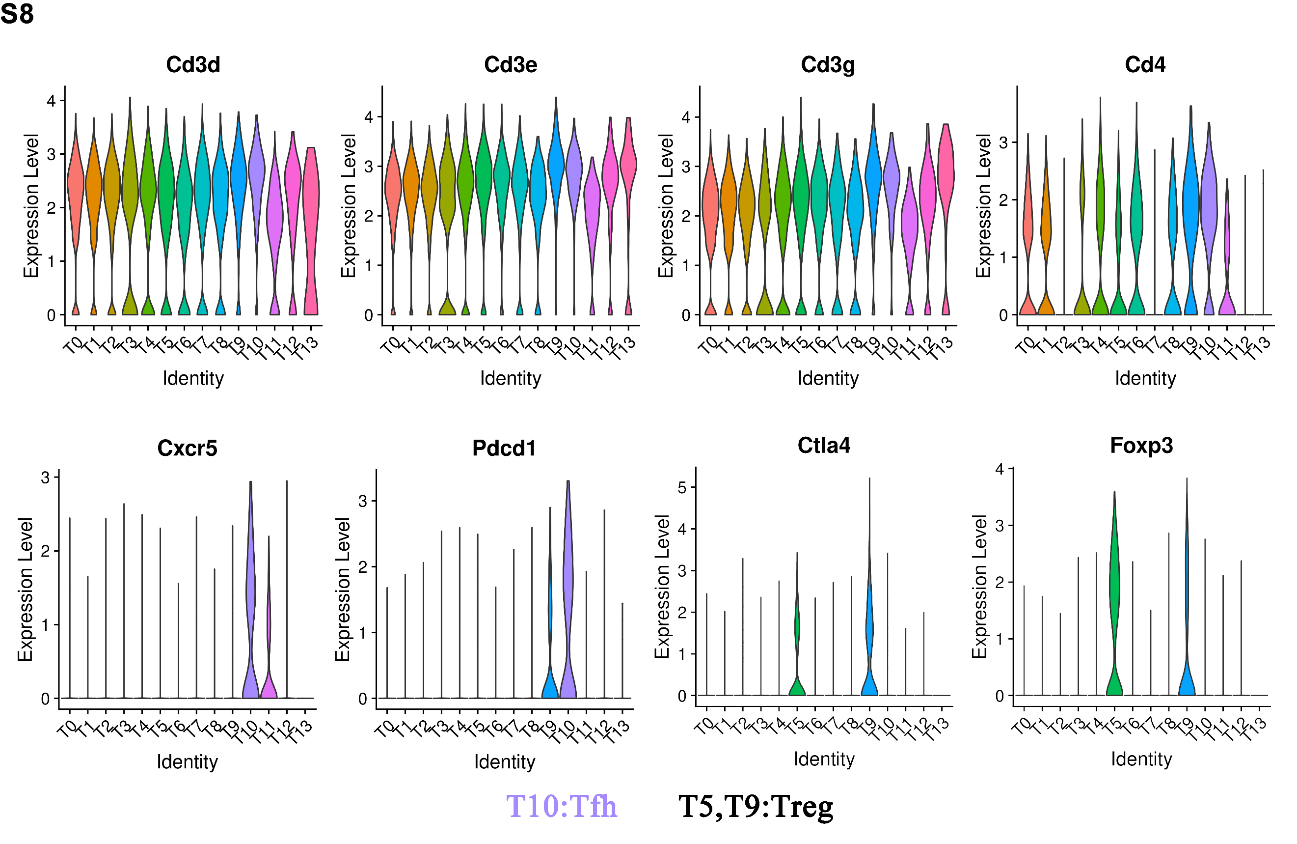
**FIG S7. Bubble plot of type I interferon-receptor-associated maker genes.** The X-axis represents the marker genes and the Y-axis represents T cell subpopulations.

**FIG S8. Violin plots of Treg and Tfh cells maker gene expression.** The title of each subplot is the marker gene name; the X-axes represent T cell subpopulations and the Y-axes represent the expression level of T cell subpopulations corresponding to the marker gene.


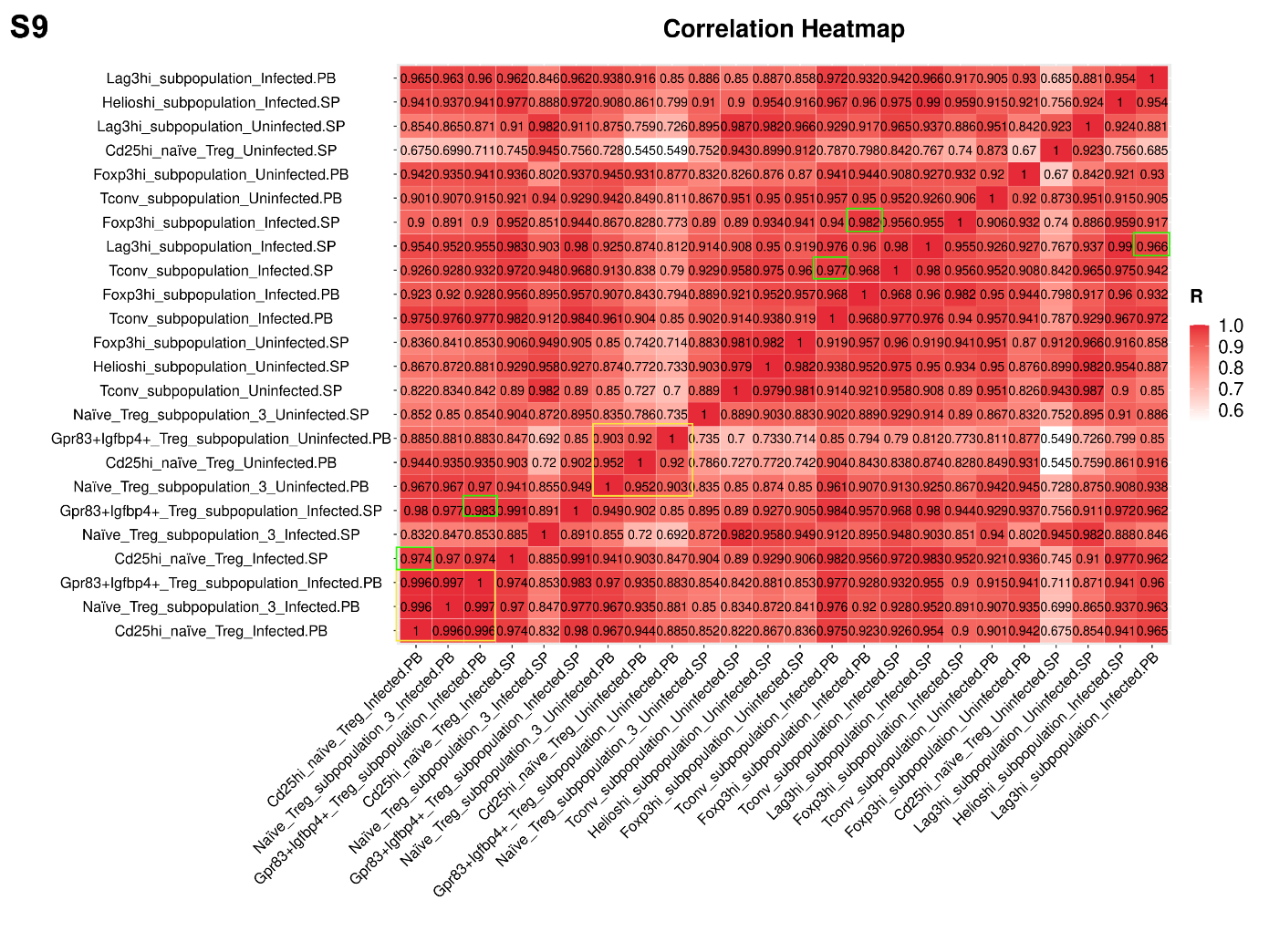


**FIG S9. Correlation analysis of different Treg subpopulations in different tissues.** The yellow rectangle represents the correlation of naïve Treg subpopulations in Infected PB and Uninfected PB, respectively. The green rectangle represents the correlation of different subpopulations in Infected PB and Infected SP.


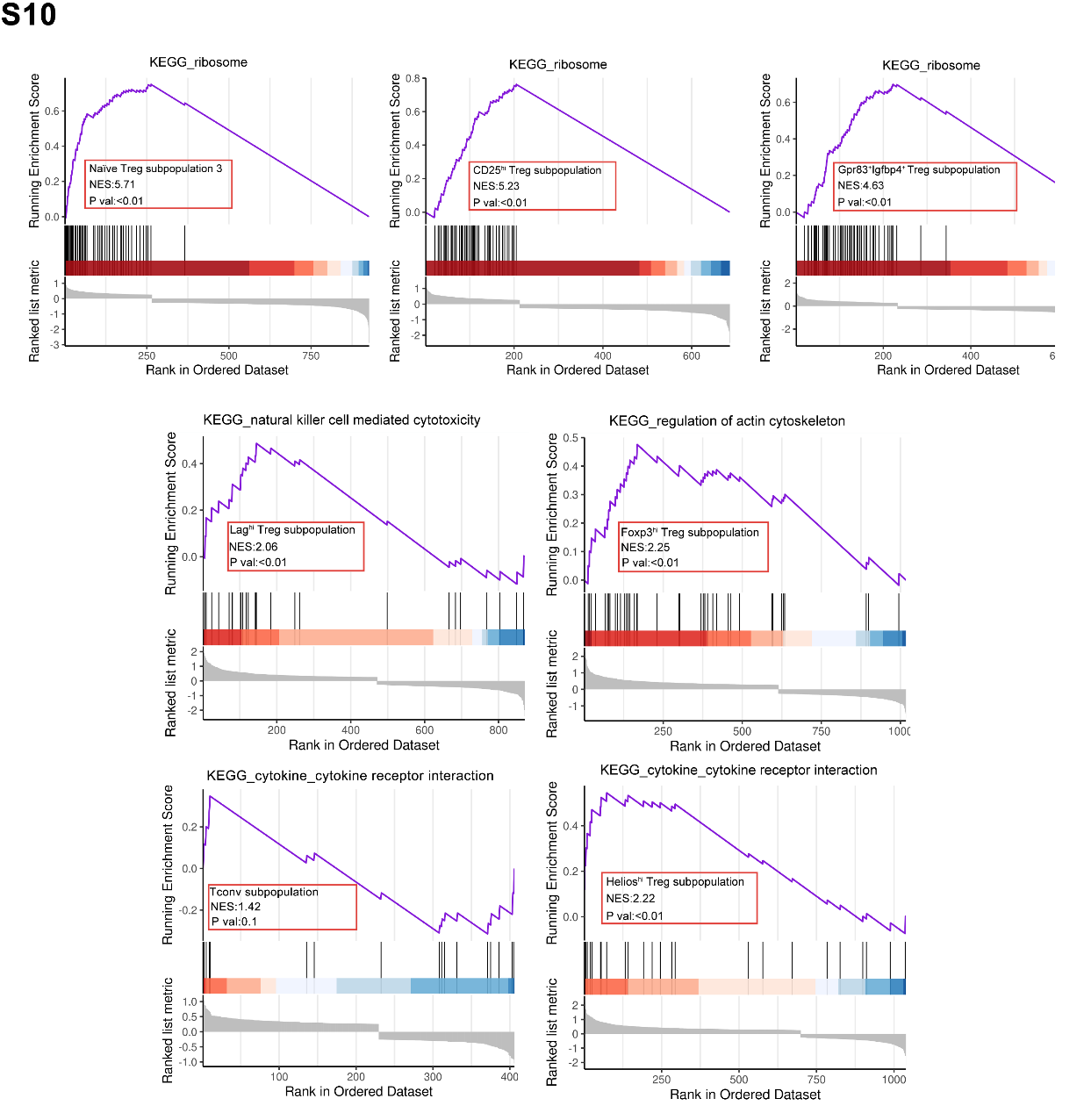


**FIG S10. Gene Set Enrichment Analysis plot.** GSEA enrichment maps of different Treg subpopulations. NES indicates enrichment fraction.


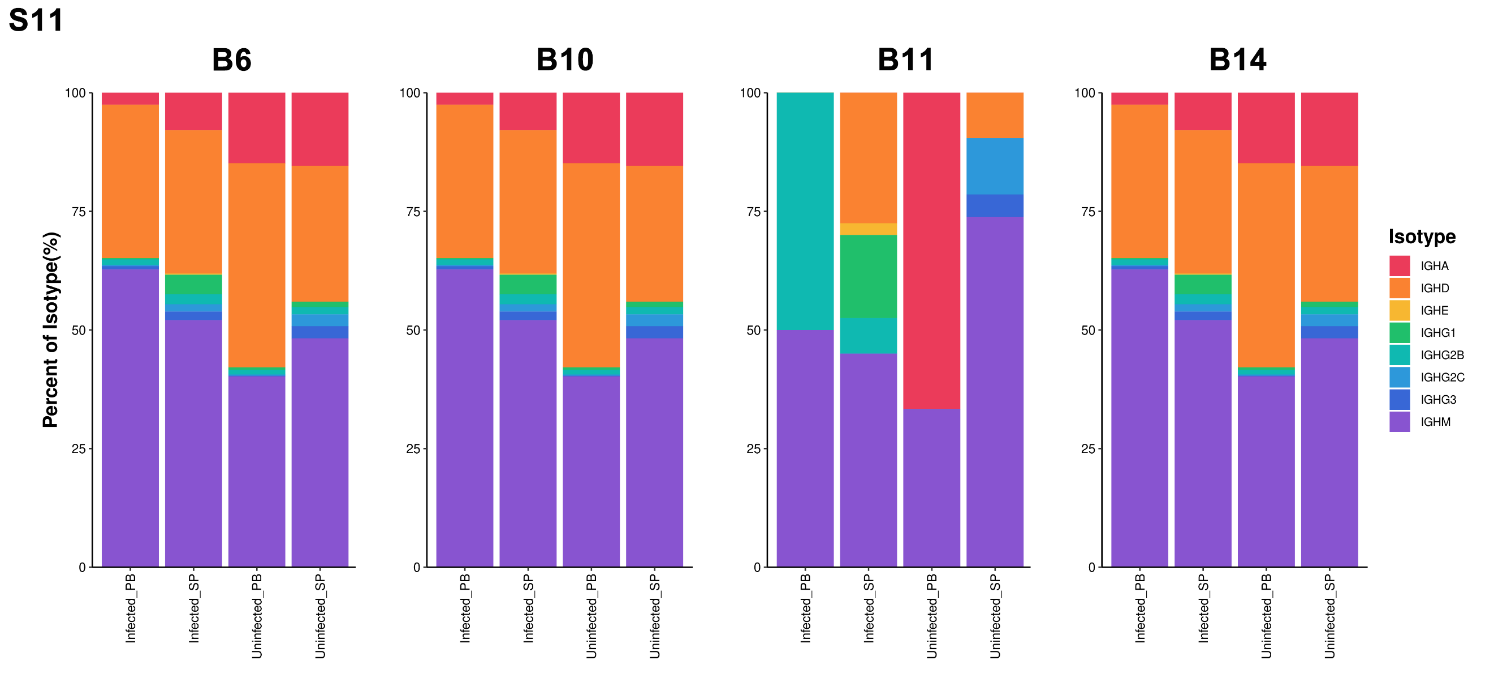


**FIG S11.** **Column graph of regulatory B cell immunoglobulin isotypes.** Different colored bars represent different immunoglobulin isotypes.


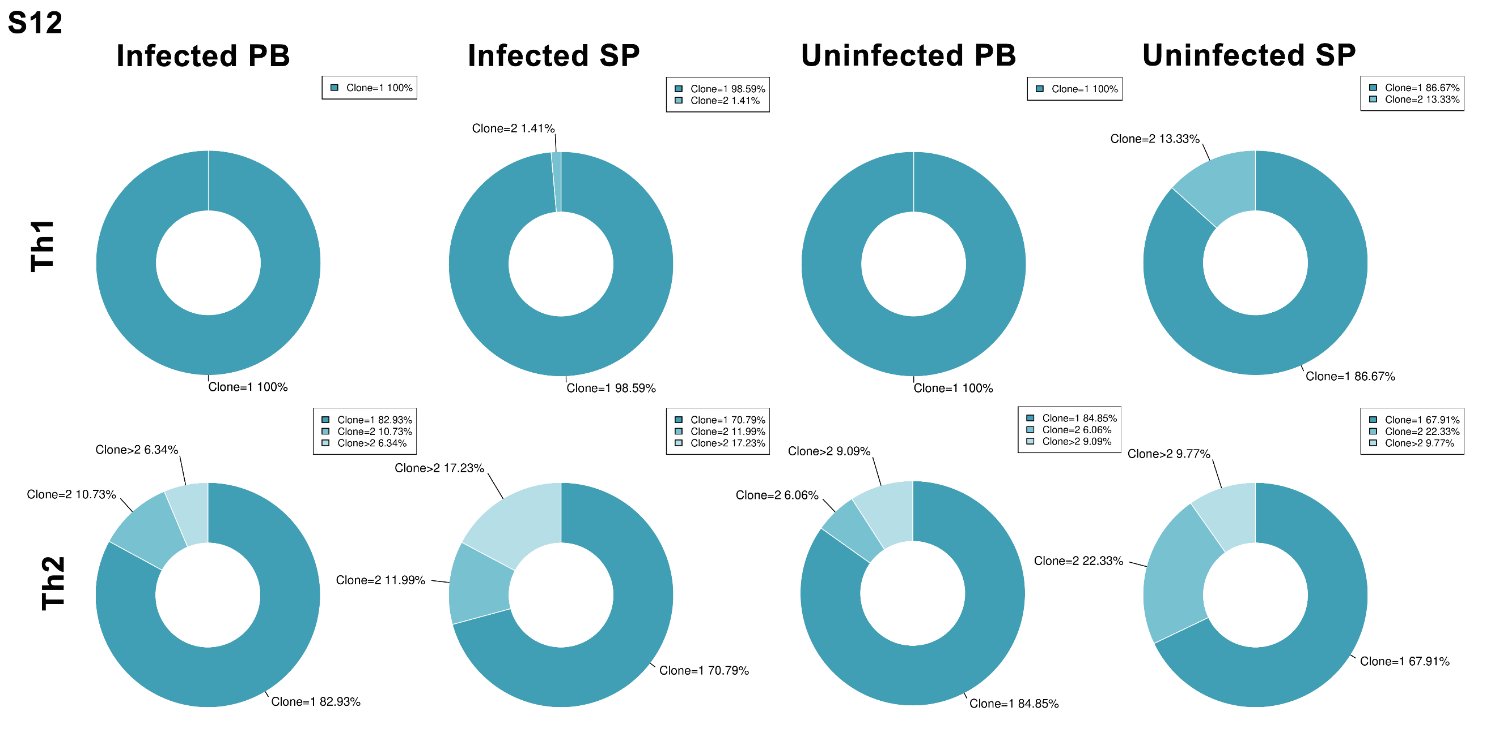


**FIG S12.** **Donut plots.** Th1/Th2 clones donut plots in different samples.


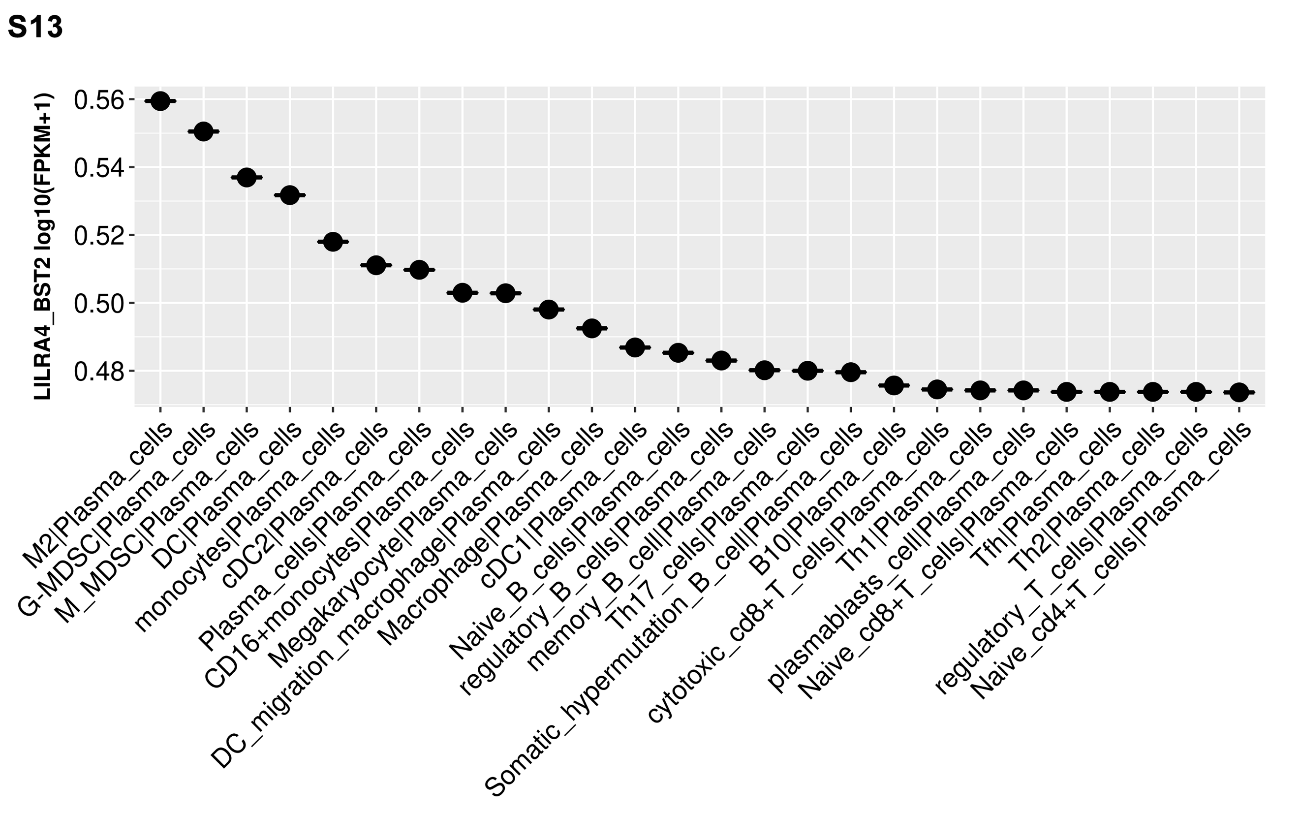


**FIG S13. Protein expression index.** Comparison of LILRA4_BST2 protein interaction pairs in plasma cells and other immune cells.


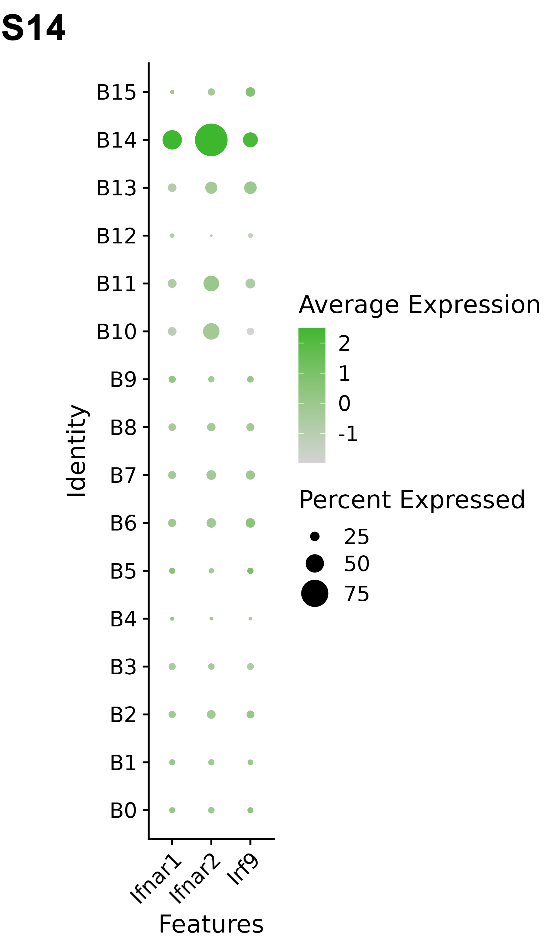


**FIG S14. Bubble plot of type I interferon-associated maker genes.** The X-axis represents the name of the marker gene and the Y-axis represents B cell subpopulations.

**TABLE S1** scRNA-seq data quality control for different samples.

| Sample | Infected PB | Infected SP | Uninfected PB | Uninfected SP |
| --- | --- | --- | --- | --- |
| Estimated Number of Cells | 15,400 | 16,765 | 7,398 | 13,735 |
| Mean Reads per Cell | 26,721 | 24,721 | 51,231 | 29,778 |
| Median Genes per Cell | 1,358 | 1,526 | 714 | 1,521 |
| Reads Mapped to Genome | 88.9% | 88.8% | 87.5% | 89.3% |
| Reads Mapped Confidently to Genome | 77.2% | 78.0% | 76.6% | 78.8% |
| Reads Mapped Confidently to Intergenic Regions | 7.1% | 5.2% | 4.5% | 6.8% |
| Reads Mapped Confidently to Intronic Regions | 10.5% | 10.2% | 13.6% | 12.5% |
| Reads Mapped Confidently to Exonic Regions | 59.7% | 62.5% | 58.4% | 59.5% |
| Reads Mapped Confidently to Transcriptome | 48.4% | 51.4% | 48.7% | 44.9% |
| Reads Mapped Antisense to Gene | 7.0% | 7.2% | 6.2% | 9.5% |
| Fraction Reads in Cells | 96.0% | 94.3% | 92.3% | 93.7% |
| Total Genes Detected | 25,187 | 26,429 | 22,054 | 25,772 |
| Number of Reads | 411,507,417 | 414,459,079 | 379,012,425 | 409,008,820 |
| Valid Barcodes | 88.5% | 89.0% | 91.7% | 83.7% |
| Sequencing Saturation | 68.8% | 57.3% | 92.1% | 61.7% |
| Q30 Bases in Barcode | 95.6% | 95.6% | 95.8% | 95.7% |
| Q30 Bases in RNA Read | 91.3% | 91.0% | 90.3% | 92.5% |
| Q30 Bases in UMI | 94.8% | 94.7% | 94.8% | 95.0% |

**TABLE S2** scRNA-seq data before and after cell filtration for different samples.

| Sample | Infected PB | Infected SP | Uninfected PB | Uninfected SP |
| --- | --- | --- | --- | --- |
| before_filter_cell_num | 15400 | 16765 | 7398 | 13735 |
| after_filter_cell_num | 13104 | 14147 | 5793 | 11820 |
| precent | 85.09% | 84.38% | 78.30% | 86.06% |

**TABLE S3** T cell subpopulation annotation.

| T0 | naïve CD4+ T cells | Cd4, Ccr7, Lef1, and Sell |
| --- | --- | --- |
| T1 | naïve CD4+ T cells | Cd4, Ccr7, Lef1, and Sell |
| T2 | naïve CD8+ T cells | Cd8a, Cd8b1, Ccr7, Lef1, and Sell |
| T3 | naïve CD4+ T cells | Cd4, Ccr7, Lef1, and Sell |
| T4 | naïve CD4+ T cells | Cd4, Ccr7, Lef1, and Sell |
| T5 | Treg | Cd4, Foxp3 |
| T6 | Th2 cell | Cd4, Gata3, Il1rl1(ST2), Ccr4 and Pparg |
| T7 | cytotoxic CD8+ T cell | Cd8a, Cd8b1, Ly6c1, and Ly6c2 |
| T8 | naïve CD4+ T cells | Cd4, Ccr7, Lef1, and Sell |
| T9 | Treg | Cd4, Foxp3 |
| T10 | Tfh | Cd4, Cxcr5 and Pdcd1 |
| T11 | others | / |
| T12 | Th1 cell | Cd4, Tbx21 (T-bet), Cxcr3, Ccl5, and Nkg7 |
| T13 | γδT cells | Cd4，Tcrg-c1 |

**TABLE S4** Cluster T3 differential gene KEGG enrichment data under Infected PB VS Uninfected PB comparison.

| pathway_name | Genes | S gene number | TS gene number | B gene number | TB gene number | pvalue |
| --- | --- | --- | --- | --- | --- | --- |
| Ribosome | Uba52;Rpl36al;Rps6;Rpl30;Rps19;Rpsa;Rpl37a;Rpl41;Rpl38;Rpl18;Rps2;Rpl37;Rps10;Rps15;Rpl39;Rps17;Rpl35a;Rps23;Rps4x;Rps18;Rpl35;Rpl27a;Rplp2;Rps15a;Rps28;Rpl10;Rps12;Rps25;Rpl27;Rpl24;Rpl31;Rps21;Rpl5;Rpl6;Mrpl33;Rpl13a;Rps29;Rplp1;Rps27;Rpl23a | 40 | 115 | 132 | 4575 | 0 |
| Oxidative phosphorylation | Ndufb5;Atp5g3;Atp5l;Atp5k;Cox7c;Uqcrfs1;Cox6c;Ndufa3;mt-Nd2;mt-Nd6 | 10 | 115 | 116 | 4575 | 0.00 |
| Parkinson disease | Cox6c;Ubb;Atp5g3;mt-Nd2;Gnai2;mt-Nd6;Ndufb5;Uqcrfs1;Ndufa3;Cox7c | 10 | 115 | 121 | 4575 | 0.00 |
| Thermogenesis | Atp5k;Ndufb5;Cox7c;Cox6c;Ndufa3;Atp5l;Actb;Uqcrfs1;Rps6;Atp5g3;mt-Nd2;mt-Nd6 | 12 | 115 | 186 | 4575 | 0.00 |
| Viral myocarditis | H2-Q4;Actb;H2-Q7;Cd40lg;H2-Q1 | 5 | 115 | 53 | 4575 | 0.01 |
| Allograft rejection | H2-Q7;Cd40lg;H2-Q4;H2-Q1 | 4 | 115 | 36 | 4575 | 0.01 |
| Autoimmune thyroid disease | H2-Q7;Cd40lg;H2-Q4;H2-Q1 | 4 | 115 | 37 | 4575 | 0.01 |
| Jak-STAT signaling pathway | Socs3;Ccnd2;Il27ra;Mcl1;Il6st;Il21r | 6 | 115 | 83 | 4575 | 0.02 |
| Th17 cell differentiation | Il27ra;Il21r;Hsp90ab1;Cd247;Nfkbia;Il6st | 6 | 115 | 84 | 4575 | 0.02 |
| Antigen processing and presentation | Hsp90ab1;H2-Q4;Tap2;H2-Q1;H2-Q7 | 5 | 115 | 63 | 4575 | 0.02 |
| Glycolysis / Gluconeogenesis | Pkm;Ldha;Gapdh;Gpi1 | 4 | 115 | 44 | 4575 | 0.02 |
| Retrograde endocannabinoid signaling | Ndufa3;Gnb2;Ndufb5;mt-Nd6;mt-Nd2;Gnai2 | 6 | 115 | 91 | 4575 | 0.03 |
| Kaposi sarcoma-associated herpesvirus infection | Ubb;H2-Q1;Zfp36;Nfkbia;H2-Q4;H2-Q7;Il6st;Gnb2 | 8 | 115 | 147 | 4575 | 0.03 |
| Human immunodeficiency virus 1 infection | Tmem173;H2-Q4;Gnb2;Nfkbia;Cd247;Gnai2;H2-Q1;H2-Q7;Tap2 | 9 | 115 | 179 | 4575 | 0.04 |
| Graft-versus-host disease | H2-Q7;H2-Q1;H2-Q4 | 3 | 115 | 37 | 4575 | 0.06 |
| Type I diabetes mellitus | H2-Q4;H2-Q1;H2-Q7 | 3 | 115 | 37 | 4575 | 0.06 |
| Alzheimer disease | Uqcrfs1;Ndufb5;Cox7c;Ndufa3;Gapdh;Atp5g3;Cox6c | 7 | 115 | 143 | 4575 | 0.07 |
| Phagosome | Tubb5;Tap2;H2-Q1;H2-Q4;Actb;H2-Q7 | 6 | 115 | 116 | 4575 | 0.07 |
| Human cytomegalovirus infection | H2-Q1;H2-Q7;H2-Q4;Tmem173;Nfkbia;Gnb2;Gnai2;Tap2 | 8 | 115 | 177 | 4575 | 0.08 |
| Cardiac muscle contraction | Cox6c;Uqcrfs1;Cox7c | 3 | 115 | 43 | 4575 | 0.09 |
| Non-alcoholic fatty liver disease (NAFLD) | Ndufa3;Cox6c;Ndufb5;Uqcrfs1;Socs3;Cox7c | 6 | 115 | 126 | 4575 | 0.10 |
| Cell adhesion molecules (CAMs) | Cd40lg;H2-Q1;H2-Q4;H2-Q7 | 4 | 115 | 78 | 4575 | 0.13 |
| Regulation of actin cytoskeleton | Arpc1b;Myl12b;Pip4k2a;Myh9;Tmsb4x;Actb | 6 | 115 | 140 | 4575 | 0.14 |
| Cellular senescence | Ccnd2;H2-Q4;Foxo1;Zfp36l2;H2-Q1;H2-Q7 | 6 | 115 | 141 | 4575 | 0.14 |
| Viral carcinogenesis | Il6st;H2-Q4;H2-Q7;H2-Q1;Pkm;Nfkbia;Ccnd2 | 7 | 115 | 173 | 4575 | 0.14 |
| Insulin resistance | Foxo1;Socs3;Nfkbia;Ogt | 4 | 115 | 81 | 4575 | 0.15 |
| IL-17 signaling pathway | Nfkbia;Jund;Hsp90ab1 | 3 | 115 | 53 | 4575 | 0.15 |
| Type II diabetes mellitus | Socs3;Pkm | 2 | 115 | 28 | 4575 | 0.16 |
| Bacterial invasion of epithelial cells | Arpc1b;Rhog;Actb | 3 | 115 | 55 | 4575 | 0.16 |
| Primary immunodeficiency | Cd40lg;Tap2 | 2 | 115 | 29 | 4575 | 0.16 |
| Epstein-Barr virus infection | Nfkbia;Ccnd2;Tap2;H2-Q1;H2-Q7;H2-Q4;Cd247 | 7 | 115 | 180 | 4575 | 0.17 |
| Huntington disease | Atp5g3;Uqcrfs1;Cox6c;Ndufa3;Ndufb5;Cox7c | 6 | 115 | 151 | 4575 | 0.18 |
| Influenza A | Socs3;Nfkbia;Actb;Dnajb1;Nxf1 | 5 | 115 | 119 | 4575 | 0.18 |
| Salmonella infection | Actb;Rhog;Arpc1b | 3 | 115 | 59 | 4575 | 0.18 |
| Apelin signaling pathway | Rps6;Klf2;Gnb2;Gnai2 | 4 | 115 | 92 | 4575 | 0.20 |
| Pyruvate metabolism | Ldha;Pkm | 2 | 115 | 33 | 4575 | 0.20 |
| Human T-cell leukemia virus 1 infection | Crtc3;Zfp36;H2-Q7;H2-Q4;H2-Q1;Nfkbia;Ccnd2 | 7 | 115 | 191 | 4575 | 0.20 |
| Spliceosome | Pcbp1;Snrpg;Srsf6;Hnrnpc;Hnrnpa3 | 5 | 115 | 125 | 4575 | 0.21 |
| Natural killer cell mediated cytotoxicity | H2-Q1;Cd247;H2-Q7;H2-Q4 | 4 | 115 | 94 | 4575 | 0.21 |
| Parathyroid hormone synthesis, secretion and action | Slc9a3r1;Gnai2;Jund | 3 | 115 | 64 | 4575 | 0.22 |
| Cytokine-cytokine receptor interaction | Il6st;Il21r;Il27ra;Cd40lg | 4 | 115 | 96 | 4575 | 0.22 |
| Osteoclast differentiation | Socs3;Junb;Jund;Nfkbia | 4 | 115 | 97 | 4575 | 0.23 |
| Tight junction | Actb;Myl12b;Slc9a3r1;Myh9 | 4 | 115 | 100 | 4575 | 0.24 |
| Glucagon signaling pathway | Pkm;Ldha;Foxo1 | 3 | 115 | 69 | 4575 | 0.25 |
| Gastric acid secretion | Gnai2;Actb | 2 | 115 | 39 | 4575 | 0.26 |
| Prostate cancer | Hsp90ab1;Foxo1;Nfkbia | 3 | 115 | 70 | 4575 | 0.26 |
| FoxO signaling pathway | Klf2;Foxo1;Ccnd2;Cdkn2d | 4 | 115 | 103 | 4575 | 0.26 |
| GABAergic synapse | Gnb2;Gnai2 | 2 | 115 | 40 | 4575 | 0.27 |
| Leukocyte transendothelial migration | Myl12b;Gnai2;Actb | 3 | 115 | 72 | 4575 | 0.27 |
| Legionellosis | Nfkbia;Eef1g | 2 | 115 | 41 | 4575 | 0.28 |
| Chagas disease (American trypanosomiasis) | Cd247;Nfkbia;Gnai2 | 3 | 115 | 74 | 4575 | 0.28 |
| Cytosolic DNA-sensing pathway | Tmem173;Nfkbia | 2 | 115 | 42 | 4575 | 0.29 |
| NF-kappa B signaling pathway | Ube2i;Cd40lg;Nfkbia | 3 | 115 | 76 | 4575 | 0.30 |
| RNA transport | Eif3f;Eif4a2;Eif3i;Ube2i;Nxf1 | 5 | 115 | 145 | 4575 | 0.30 |
| Morphine addiction | Gnai2;Gnb2 | 2 | 115 | 44 | 4575 | 0.30 |
| Central carbon metabolism in cancer | Ldha;Pkm | 2 | 115 | 44 | 4575 | 0.30 |
| RIG-I-like receptor signaling pathway | Nfkbia;Tmem173 | 2 | 115 | 45 | 4575 | 0.31 |
| Asthma | Cd40lg | 1 | 115 | 15 | 4575 | 0.32 |
| HIF-1 signaling pathway | Gapdh;Ldha;Rps6 | 3 | 115 | 79 | 4575 | 0.32 |
| Toxoplasmosis | Nfkbia;Cd40lg;Gnai2 | 3 | 115 | 79 | 4575 | 0.32 |
| Human papillomavirus infection | Foxo1;Pkm;H2-Q4;H2-Q7;Ccnd2;H2-Q1;Slc9a3r1 | 7 | 115 | 223 | 4575 | 0.33 |
| Relaxin signaling pathway | Gnb2;Gnai2;Nfkbia | 3 | 115 | 83 | 4575 | 0.35 |
| Prolactin signaling pathway | Socs3;Ccnd2 | 2 | 115 | 49 | 4575 | 0.35 |
| Circadian entrainment | Gnb2;Gnai2 | 2 | 115 | 49 | 4575 | 0.35 |
| Starch and sucrose metabolism | Gpi1 | 1 | 115 | 17 | 4575 | 0.35 |
| TNF signaling pathway | Socs3;Junb;Nfkbia | 3 | 115 | 84 | 4575 | 0.35 |
| Adipocytokine signaling pathway | Socs3;Nfkbia | 2 | 115 | 50 | 4575 | 0.36 |
| Serotonergic synapse | Gnb2;Gnai2 | 2 | 115 | 50 | 4575 | 0.36 |
| Oxytocin signaling pathway | Eef2;Gnai2;Actb | 3 | 115 | 85 | 4575 | 0.36 |
| Other types of O-glycan biosynthesis | Ogt | 1 | 115 | 18 | 4575 | 0.37 |
| Gap junction | Gnai2;Tubb5 | 2 | 115 | 52 | 4575 | 0.38 |
| Platelet activation | Actb;Gnai2;Myl12b | 3 | 115 | 90 | 4575 | 0.40 |
| T cell receptor signaling pathway | Nfkbia;Cd247;Cd40lg | 3 | 115 | 90 | 4575 | 0.40 |
| Glutamatergic synapse | Gnai2;Gnb2 | 2 | 115 | 54 | 4575 | 0.40 |
| NOD-like receptor signaling pathway | Nfkbia;Txnip;Hsp90ab1;Tmem173 | 4 | 115 | 128 | 4575 | 0.40 |
| Mitophagy - animal | Tomm7;Ubb | 2 | 115 | 55 | 4575 | 0.40 |
| Fluid shear stress and atherosclerosis | Klf2;Actb;Hsp90ab1 | 3 | 115 | 95 | 4575 | 0.43 |
| Endocytosis | Sh3glb1;H2-Q4;H2-Q7;Ubb;Arpc1b;H2-Q1 | 6 | 115 | 212 | 4575 | 0.44 |
| Pentose phosphate pathway | Gpi1 | 1 | 115 | 23 | 4575 | 0.44 |
| ABC transporters | Tap2 | 1 | 115 | 23 | 4575 | 0.44 |
| Malaria | Cd40lg | 1 | 115 | 24 | 4575 | 0.46 |
| MicroRNAs in cancer | Mcl1;Ccnd2;Ube2i | 3 | 115 | 100 | 4575 | 0.46 |
| Herpes simplex virus 1 infection | Srsf6;H2-Q1;H2-Q7;Tap2;Nxf1;Socs3;Nfkbia;Tmem173;H2-Q4 | 9 | 115 | 335 | 4575 | 0.47 |
| Cocaine addiction | Gnai2 | 1 | 115 | 26 | 4575 | 0.49 |
| Progesterone-mediated oocyte maturation | Hsp90ab1;Gnai2 | 2 | 115 | 66 | 4575 | 0.50 |
| Hedgehog signaling pathway | Ccnd2 | 1 | 115 | 27 | 4575 | 0.50 |
| Protein processing in endoplasmic reticulum | Dnajb1;Dnaja1;Ppp1r15a;Hsp90ab1 | 4 | 115 | 146 | 4575 | 0.50 |
| Cholinergic synapse | Gnb2;Gnai2 | 2 | 115 | 67 | 4575 | 0.51 |
| Insulin signaling pathway | Socs3;Foxo1;Rps6 | 3 | 115 | 107 | 4575 | 0.51 |
| Intestinal immune network for IgA production | Cd40lg | 1 | 115 | 29 | 4575 | 0.52 |
| Propanoate metabolism | Ldha | 1 | 115 | 29 | 4575 | 0.52 |
| Th1 and Th2 cell differentiation | Nfkbia;Cd247 | 2 | 115 | 70 | 4575 | 0.53 |
| Regulation of lipolysis in adipocytes | Gnai2 | 1 | 115 | 30 | 4575 | 0.54 |
| PI3K-Akt signaling pathway | Gnb2;Ccnd2;Hsp90ab1;Mcl1;Rps6 | 5 | 115 | 196 | 4575 | 0.55 |
| Estrogen signaling pathway | Gnai2;Hsp90ab1 | 2 | 115 | 74 | 4575 | 0.56 |
| Focal adhesion | Myl12b;Ccnd2;Actb | 3 | 115 | 116 | 4575 | 0.56 |
| Arrhythmogenic right ventricular cardiomyopathy (ARVC) | Actb | 1 | 115 | 33 | 4575 | 0.57 |
| Ferroptosis | Pcbp1 | 1 | 115 | 33 | 4575 | 0.57 |
| Renin secretion | Gnai2 | 1 | 115 | 33 | 4575 | 0.57 |
| Chemokine signaling pathway | Nfkbia;Gnai2;Gnb2 | 3 | 115 | 119 | 4575 | 0.58 |
| Apoptosis | Mcl1;Actb;Nfkbia | 3 | 115 | 119 | 4575 | 0.58 |
| Vasopressin-regulated water reabsorption | Arhgdia | 1 | 115 | 35 | 4575 | 0.59 |
| Cysteine and methionine metabolism | Ldha | 1 | 115 | 36 | 4575 | 0.60 |
| Long-term depression | Gnai2 | 1 | 115 | 36 | 4575 | 0.60 |
| Hypertrophic cardiomyopathy (HCM) | Actb | 1 | 115 | 37 | 4575 | 0.61 |
| Inflammatory bowel disease (IBD) | Il21r | 1 | 115 | 37 | 4575 | 0.61 |
| Ubiquitin mediated proteolysis | Herc1;Ube2i;Socs3 | 3 | 115 | 126 | 4575 | 0.62 |
| Hippo signaling pathway | Ccnd2;Actb | 2 | 115 | 84 | 4575 | 0.63 |
| Dilated cardiomyopathy (DCM) | Actb | 1 | 115 | 39 | 4575 | 0.63 |
| Dopaminergic synapse | Gnai2;Gnb2 | 2 | 115 | 86 | 4575 | 0.64 |
| Purine metabolism | Nme1;Pkm | 2 | 115 | 87 | 4575 | 0.65 |
| Drug metabolism - other enzymes | Nme1 | 1 | 115 | 41 | 4575 | 0.65 |
| Amino sugar and nucleotide sugar metabolism | Gpi1 | 1 | 115 | 42 | 4575 | 0.66 |
| AMPK signaling pathway | Foxo1;Eef2 | 2 | 115 | 90 | 4575 | 0.67 |
| Pyrimidine metabolism | Nme1 | 1 | 115 | 43 | 4575 | 0.67 |
| Proteasome | Psmb8 | 1 | 115 | 43 | 4575 | 0.67 |
| Nucleotide excision repair | Xpa | 1 | 115 | 43 | 4575 | 0.67 |
| Cushing syndrome | Gnai2;Aip | 2 | 115 | 91 | 4575 | 0.67 |
| Thyroid hormone signaling pathway | Actb;Foxo1 | 2 | 115 | 91 | 4575 | 0.67 |
| Longevity regulating pathway - multiple species | Foxo1 | 1 | 115 | 46 | 4575 | 0.69 |
| Pertussis | Gnai2 | 1 | 115 | 47 | 4575 | 0.70 |
| Axon guidance | Gnai2;Myl12b | 2 | 115 | 97 | 4575 | 0.71 |
| Neurotrophin signaling pathway | Arhgdia;Nfkbia | 2 | 115 | 98 | 4575 | 0.71 |
| Pathways in cancer | Ccnd2;Gnai2;Nfkbia;Hsp90ab1;Il6st;Gnb2;Foxo1 | 7 | 115 | 329 | 4575 | 0.73 |
| Adherens junction | Actb | 1 | 115 | 52 | 4575 | 0.74 |
| Melanogenesis | Gnai2 | 1 | 115 | 53 | 4575 | 0.74 |
| Cell cycle | Ccnd2;Cdkn2d | 2 | 115 | 105 | 4575 | 0.75 |
| Leishmaniasis | Nfkbia | 1 | 115 | 54 | 4575 | 0.75 |
| Alcoholism | Gnai2;Gnb2 | 2 | 115 | 106 | 4575 | 0.75 |
| cAMP signaling pathway | Gnai2;Nfkbia | 2 | 115 | 106 | 4575 | 0.75 |
| Measles | Ccnd2;Nfkbia | 2 | 115 | 106 | 4575 | 0.75 |
| EGFR tyrosine kinase inhibitor resistance | Rps6 | 1 | 115 | 56 | 4575 | 0.76 |
| p53 signaling pathway | Ccnd2 | 1 | 115 | 56 | 4575 | 0.76 |
| Transcriptional misregulation in cancer | Foxo1;Ccnd2 | 2 | 115 | 112 | 4575 | 0.78 |
| Hepatitis C | Nfkbia;Socs3 | 2 | 115 | 112 | 4575 | 0.78 |
| Platinum drug resistance | Xpa | 1 | 115 | 60 | 4575 | 0.79 |
| Inositol phosphate metabolism | Pip4k2a | 1 | 115 | 61 | 4575 | 0.79 |
| Vascular smooth muscle contraction | Ramp1 | 1 | 115 | 64 | 4575 | 0.81 |
| Systemic lupus erythematosus | Cd40lg | 1 | 115 | 65 | 4575 | 0.81 |
| AGE-RAGE signaling pathway in diabetic complications | Foxo1 | 1 | 115 | 66 | 4575 | 0.82 |
| Toll-like receptor signaling pathway | Nfkbia | 1 | 115 | 67 | 4575 | 0.82 |
| Chronic myeloid leukemia | Nfkbia | 1 | 115 | 67 | 4575 | 0.82 |
| Proteoglycans in cancer | Actb;Rps6 | 2 | 115 | 123 | 4575 | 0.82 |
| Rap1 signaling pathway | Gnai2;Actb | 2 | 115 | 125 | 4575 | 0.83 |
| B cell receptor signaling pathway | Nfkbia | 1 | 115 | 69 | 4575 | 0.83 |
| Small cell lung cancer | Nfkbia | 1 | 115 | 69 | 4575 | 0.83 |
| Fc gamma R-mediated phagocytosis | Arpc1b | 1 | 115 | 70 | 4575 | 0.83 |
| Longevity regulating pathway | Foxo1 | 1 | 115 | 71 | 4575 | 0.84 |
| Signaling pathways regulating pluripotency of stem cells | Il6st | 1 | 115 | 71 | 4575 | 0.84 |
| Ribosome biogenesis in eukaryotes | Nxf1 | 1 | 115 | 73 | 4575 | 0.85 |
| C-type lectin receptor signaling pathway | Nfkbia | 1 | 115 | 77 | 4575 | 0.86 |
| mRNA surveillance pathway | Nxf1 | 1 | 115 | 79 | 4575 | 0.87 |
| Phosphatidylinositol signaling system | Pip4k2a | 1 | 115 | 80 | 4575 | 0.87 |
| Adrenergic signaling in cardiomyocytes | Gnai2 | 1 | 115 | 82 | 4575 | 0.88 |
| Wnt signaling pathway | Ccnd2 | 1 | 115 | 88 | 4575 | 0.90 |
| Sphingolipid signaling pathway | Gnai2 | 1 | 115 | 98 | 4575 | 0.92 |
| cGMP-PKG signaling pathway | Gnai2 | 1 | 115 | 98 | 4575 | 0.92 |
| mTOR signaling pathway | Rps6 | 1 | 115 | 114 | 4575 | 0.95 |
| Autophagy - animal | Sh3glb1 | 1 | 115 | 115 | 4575 | 0.95 |
| Necroptosis | Hsp90ab1 | 1 | 115 | 115 | 4575 | 0.95 |
| Hepatocellular carcinoma | Actb | 1 | 115 | 117 | 4575 | 0.95 |
| Ras signaling pathway | Gnb2 | 1 | 115 | 127 | 4575 | 0.96 |
| Hepatitis B | Nfkbia | 1 | 115 | 127 | 4575 | 0.96 |
| MAPK signaling pathway | Jund | 1 | 115 | 182 | 4575 | 0.99 |

**TABLE S5** Cell number and percentage of Treg subpopulations in different samples.

| Clusters | 0 | 1 | 2 | 3 | 4 | 5 | 6 |
| --- | --- | --- | --- | --- | --- | --- | --- |
| Infected PB | 18(7.86%) | 44(21.36%) | 99（48.29%） | 133（67.86%） | 138（73.4%） | 0（0%） | 108（78.83%） |
| Infected SP | 133(58.08%) | 50(24.27%) | 46（22.44%） | 25（12.76%） | 32（17.02%） | 96（63.16%） | 27（19.71%） |
| Uninfected PB | 0(0%) | 9(4.37%) | 20（9.76%） | 34（17.35%） | 12（6.38%） | 0（0%） | 2（1.46%） |
| Uninfected SP | 78(34.06%) | 103（50%） | 40（19.51%） | 4（2.04%） | 6（3.19%） | 56（36.84%） | 0（0%） |

**TABLE S6** The number of clones of activated T cell subpopulations in different samples.

| clonotype | Infected PB_cytotoxic_cd8+T_cells | Infected SP_cytotoxic_cd8+T_cells | Uninfected PB_cytotoxic_cd8+T_cells | Uninfected SP_cytotoxic_cd8+T_cells |
| --- | --- | --- | --- | --- |
| Clone=1 | 84.72% | 75.94% | 94.12% | 75% |
| Clone=2 | 15.28% | 16.54% | 5.88% | 18.18% |
| Clone>2 | / | 7.52% | / | 6.82% |
|  |  |  |  |  |
| clonotype | Infected PB_gama_delta_T_cells | Infected SP_gama_delta_T_cells | Uninfected PB_gama_delta_T_cells | Uninfected SP_gama_delta_T_cells |
| Clone=1 | 100% | 100% | / | 100% |
| Clone=2 | / | / | / | / |
|  |  |  |  |  |
| clonotype | Infected PB_regulatory_T_cells | Infected SP_regulatory_T_cells | Uninfected PB_regulatory_T_cells | Uninfected SP_regulatory_T_cells |
| Clone=1 | 96.38% | 86.15% | 100% | 98% |
| Clone=2 | 2.98% | 9.57% | / | 10.75% |
| Clone>2 | 0.64% | 4.28% | / | 2.15% |
|  |  |  |  |  |
| clonotype | Infected PB_Tfh | Infected SP_Tfh | Uninfected PB_Tfh | Uninfected SP_Tfh |
| Clone=1 | 89.47% | 64.66% | / | 58.62% |
| Clone=2 | 10.53% | 23.29% | / | 20.69% |
| Clone>2 | / | 12.05% | / | 20.69% |
|  |  |  |  |  |
| clonotype | Infected PB_Th1 | Infected SP_Th1 | Uninfected PB_Th1 | Uninfected SP_Th1 |
| Clone=1 | 100% | 98.59% | 100% | 86.67% |
| Clone=2 | / | 1.41% | / | 13.33% |
|  |  |  |  |  |
| clonotype | Infected PB_Th2 | Infected SP_Th2 | Uninfected PB_Th2 | Uninfected SP_Th2 |
| Clone=1 | 82.93% | 70.79% | 84.85% | 67.91% |
| Clone=2 | 10.73% | 11.99% | 6.06% | 22.33% |
| Clone>2 | 6.34% | 17.23% | 9.09% | 9.77% |

**TABLE S7** The number of clones of activated B cell subpopulations in different samples.

| clonotype | Infected PB_B10 | Infected SP_B10 | Uninfected PB_B10 | Uninfected SP_B10 |
| --- | --- | --- | --- | --- |
| Clone=1 | 100% | 66.67% | 100% | 86.36% |
| Clone=2 | / | 3.92% | / | 4.55% |
| Clone>2 | / | 29.41% | / | 9.09% |
|  |  |  |  |  |
| clonotype | Infected PB_memory_B_cell | Infected SP_memory_B_cell | Uninfected PB_memory_B_cell | Uninfected SP_memory_B_cell |
| Clone=1 | 74.06% | 78.85% | 30.84% | 85.84% |
| Clone=2 | 10.67% | 5.02% | 2.09% | 8.85% |
| Clone>2 | 15.26% | 16.13% | 67.07% | 5.31% |
|  |  |  |  |  |
| clonotype | Infected PB_Plasma_cells | Infected SP_Plasma_cells | Uninfected PB_Plasma_cells | Uninfected SP_Plasma_cells |
| Clone=1 | 100% | 100% | / | 69.23% |
| Clone=2 | / | / | / | 5.13% |
| Clone>2 | / | / | / | 25.64% |
|  |  |  |  |  |
| clonotype | Infected PB_plasmablasts_cell | Infected SP_plasmablasts_cell | Uninfected PB_plasmablasts_cell | Uninfected SP_plasmablasts_cell |
| Clone=1 | 100% | 81.69% | 73.58% | 67.12% |
| Clone=2 | / | 10.33% | 18.87% | 15.53% |
| Clone>2 | / | 7.98% | 7.55% | 17.35% |
|  |  |  |  |  |
| clonotype | Infected PB_regulatory_B_cells. | Infected SP_regulatory_B_cells. | Uninfected PB_regulatory_B_cells | Uninfected SP_regulatory_B_cells |
| Clone=1 | 55.95% | 64.88% | 41.97% | 47.71% |
| Clone=2 | 22.70% | 8.93% | 5.76% | 6.71% |
| Clone>2 | 21.35% | 26.19% | 52.27% | 45.58% |
|  |  |  |  |  |
| clonotype | Infected PB_Somatic_hypermutation_B_cell | Infected SP_Somatic_hypermutation_B_cell | Uninfected PB_Somatic_hypermutation_B_cell | Uninfected SP_Somatic_hypermutation_B_cell |
| Clone=1 | 100% | 81.03% | / | 61.54% |
| Clone=2 | / | 1.15% | / | 38.46% |
| Clone>2 | / | 17.82% | / | / |

**TABLE S8** Treg-Tfh-B cell subpopulations cell interaction index.

| SOURCE | TARGET | count |
| --- | --- | --- |
| Naive_B_cells | Naive_B_cells | 10 |
| Naive_B_cells | regulatory_B_cells | 8 |
| Naive_B_cells | memory_B_cell | 28 |
| Naive_B_cells | Somatic_hypermutation_B_cell | 18 |
| Naive_B_cells | Plasma_cells | 38 |
| Naive_B_cells | plasmablasts_cell | 13 |
| Naive_B_cells | B10 | 29 |
| Naive_B_cells | Memory_Tfh | 24 |
| Naive_B_cells | Tfh17 | 24 |
| Naive_B_cells | Tfh2 | 22 |
| Naive_B_cells | Cd25hi_naÃ¯ve_Treg | 21 |
| Naive_B_cells | NaÃ¯ve_Treg | 16 |
| Naive_B_cells | Gpr83+Igfbp4+_Treg_cells | 19 |
| Naive_B_cells | Tconv | 22 |
| Naive_B_cells | Helioshi_subpopulation | 25 |
| Naive_B_cells | Foxp3hi_subpopulation | 28 |
| Naive_B_cells | Lag3hi_subpopulation | 24 |
| regulatory_B_cells | Naive_B_cells | 8 |
| regulatory_B_cells | regulatory_B_cells | 1 |
| regulatory_B_cells | memory_B_cell | 10 |
| regulatory_B_cells | Somatic_hypermutation_B_cell | 10 |
| regulatory_B_cells | Plasma_cells | 18 |
| regulatory_B_cells | plasmablasts_cell | 7 |
| regulatory_B_cells | B10 | 11 |
| regulatory_B_cells | Memory_Tfh | 10 |
| regulatory_B_cells | Tfh17 | 11 |
| regulatory_B_cells | Tfh2 | 10 |
| regulatory_B_cells | Cd25hi_naÃ¯ve_Treg | 9 |
| regulatory_B_cells | NaÃ¯ve_Treg | 7 |
| regulatory_B_cells | Gpr83+Igfbp4+_Treg_cells | 6 |
| regulatory_B_cells | Tconv | 8 |
| regulatory_B_cells | Helioshi_subpopulation | 12 |
| regulatory_B_cells | Foxp3hi_subpopulation | 12 |
| regulatory_B_cells | Lag3hi_subpopulation | 12 |
| memory_B_cell | Naive_B_cells | 28 |
| memory_B_cell | regulatory_B_cells | 10 |
| memory_B_cell | memory_B_cell | 17 |
| memory_B_cell | Somatic_hypermutation_B_cell | 26 |
| memory_B_cell | Plasma_cells | 42 |
| memory_B_cell | plasmablasts_cell | 10 |
| memory_B_cell | B10 | 39 |
| memory_B_cell | Memory_Tfh | 25 |
| memory_B_cell | Tfh17 | 30 |
| memory_B_cell | Tfh2 | 29 |
| memory_B_cell | Cd25hi_naÃ¯ve_Treg | 17 |
| memory_B_cell | NaÃ¯ve_Treg | 12 |
| memory_B_cell | Gpr83+Igfbp4+_Treg_cells | 15 |
| memory_B_cell | Tconv | 21 |
| memory_B_cell | Helioshi_subpopulation | 34 |
| memory_B_cell | Foxp3hi_subpopulation | 36 |
| memory_B_cell | Lag3hi_subpopulation | 36 |
| Somatic_hypermutation_B_cell | Naive_B_cells | 18 |
| Somatic_hypermutation_B_cell | regulatory_B_cells | 10 |
| Somatic_hypermutation_B_cell | memory_B_cell | 26 |
| Somatic_hypermutation_B_cell | Somatic_hypermutation_B_cell | 11 |
| Somatic_hypermutation_B_cell | Plasma_cells | 43 |
| Somatic_hypermutation_B_cell | plasmablasts_cell | 16 |
| Somatic_hypermutation_B_cell | B10 | 34 |
| Somatic_hypermutation_B_cell | Memory_Tfh | 31 |
| Somatic_hypermutation_B_cell | Tfh17 | 33 |
| Somatic_hypermutation_B_cell | Tfh2 | 30 |
| Somatic_hypermutation_B_cell | Cd25hi_naÃ¯ve_Treg | 29 |
| Somatic_hypermutation_B_cell | NaÃ¯ve_Treg | 21 |
| Somatic_hypermutation_B_cell | Gpr83+Igfbp4+_Treg_cells | 26 |
| Somatic_hypermutation_B_cell | Tconv | 26 |
| Somatic_hypermutation_B_cell | Helioshi_subpopulation | 36 |
| Somatic_hypermutation_B_cell | Foxp3hi_subpopulation | 34 |
| Somatic_hypermutation_B_cell | Lag3hi_subpopulation | 36 |
| Plasma_cells | Naive_B_cells | 38 |
| Plasma_cells | regulatory_B_cells | 18 |
| Plasma_cells | memory_B_cell | 42 |
| Plasma_cells | Somatic_hypermutation_B_cell | 43 |
| Plasma_cells | Plasma_cells | 34 |
| Plasma_cells | plasmablasts_cell | 28 |
| Plasma_cells | B10 | 62 |
| Plasma_cells | Memory_Tfh | 35 |
| Plasma_cells | Tfh17 | 42 |
| Plasma_cells | Tfh2 | 43 |
| Plasma_cells | Cd25hi_naÃ¯ve_Treg | 29 |
| Plasma_cells | NaÃ¯ve_Treg | 20 |
| Plasma_cells | Gpr83+Igfbp4+_Treg_cells | 29 |
| Plasma_cells | Tconv | 29 |
| Plasma_cells | Helioshi_subpopulation | 58 |
| Plasma_cells | Foxp3hi_subpopulation | 47 |
| Plasma_cells | Lag3hi_subpopulation | 54 |
| plasmablasts_cell | Naive_B_cells | 13 |
| plasmablasts_cell | regulatory_B_cells | 7 |
| plasmablasts_cell | memory_B_cell | 10 |
| plasmablasts_cell | Somatic_hypermutation_B_cell | 16 |
| plasmablasts_cell | Plasma_cells | 28 |
| plasmablasts_cell | plasmablasts_cell | 2 |
| plasmablasts_cell | B10 | 23 |
| plasmablasts_cell | Memory_Tfh | 12 |
| plasmablasts_cell | Tfh17 | 15 |
| plasmablasts_cell | Tfh2 | 14 |
| plasmablasts_cell | Cd25hi_naÃ¯ve_Treg | 8 |
| plasmablasts_cell | NaÃ¯ve_Treg | 5 |
| plasmablasts_cell | Gpr83+Igfbp4+_Treg_cells | 9 |
| plasmablasts_cell | Tconv | 12 |
| plasmablasts_cell | Helioshi_subpopulation | 24 |
| plasmablasts_cell | Foxp3hi_subpopulation | 25 |
| plasmablasts_cell | Lag3hi_subpopulation | 21 |
| B10 | Naive_B_cells | 29 |
| B10 | regulatory_B_cells | 11 |
| B10 | memory_B_cell | 39 |
| B10 | Somatic_hypermutation_B_cell | 34 |
| B10 | Plasma_cells | 62 |
| B10 | plasmablasts_cell | 23 |
| B10 | B10 | 36 |
| B10 | Memory_Tfh | 39 |
| B10 | Tfh17 | 48 |
| B10 | Tfh2 | 51 |
| B10 | Cd25hi_naÃ¯ve_Treg | 35 |
| B10 | NaÃ¯ve_Treg | 24 |
| B10 | Gpr83+Igfbp4+_Treg_cells | 37 |
| B10 | Tconv | 33 |
| B10 | Helioshi_subpopulation | 61 |
| B10 | Foxp3hi_subpopulation | 58 |
| B10 | Lag3hi_subpopulation | 60 |
| Memory_Tfh | Naive_B_cells | 24 |
| Memory_Tfh | regulatory_B_cells | 10 |
| Memory_Tfh | memory_B_cell | 25 |
| Memory_Tfh | Somatic_hypermutation_B_cell | 31 |
| Memory_Tfh | Plasma_cells | 35 |
| Memory_Tfh | plasmablasts_cell | 12 |
| Memory_Tfh | B10 | 39 |
| Memory_Tfh | Memory_Tfh | 7 |
| Memory_Tfh | Tfh17 | 22 |
| Memory_Tfh | Tfh2 | 24 |
| Memory_Tfh | Cd25hi_naÃ¯ve_Treg | 15 |
| Memory_Tfh | NaÃ¯ve_Treg | 6 |
| Memory_Tfh | Gpr83+Igfbp4+_Treg_cells | 16 |
| Memory_Tfh | Tconv | 15 |
| Memory_Tfh | Helioshi_subpopulation | 33 |
| Memory_Tfh | Foxp3hi_subpopulation | 26 |
| Memory_Tfh | Lag3hi_subpopulation | 33 |
| Tfh17 | Naive_B_cells | 24 |
| Tfh17 | regulatory_B_cells | 11 |
| Tfh17 | memory_B_cell | 30 |
| Tfh17 | Somatic_hypermutation_B_cell | 33 |
| Tfh17 | Plasma_cells | 42 |
| Tfh17 | plasmablasts_cell | 15 |
| Tfh17 | B10 | 48 |
| Tfh17 | Memory_Tfh | 22 |
| Tfh17 | Tfh17 | 16 |
| Tfh17 | Tfh2 | 29 |
| Tfh17 | Cd25hi_naÃ¯ve_Treg | 22 |
| Tfh17 | NaÃ¯ve_Treg | 12 |
| Tfh17 | Gpr83+Igfbp4+_Treg_cells | 22 |
| Tfh17 | Tconv | 17 |
| Tfh17 | Helioshi_subpopulation | 42 |
| Tfh17 | Foxp3hi_subpopulation | 33 |
| Tfh17 | Lag3hi_subpopulation | 40 |
| Tfh2 | Naive_B_cells | 22 |
| Tfh2 | regulatory_B_cells | 10 |
| Tfh2 | memory_B_cell | 29 |
| Tfh2 | Somatic_hypermutation_B_cell | 30 |
| Tfh2 | Plasma_cells | 43 |
| Tfh2 | plasmablasts_cell | 14 |
| Tfh2 | B10 | 51 |
| Tfh2 | Memory_Tfh | 24 |
| Tfh2 | Tfh17 | 29 |
| Tfh2 | Tfh2 | 16 |
| Tfh2 | Cd25hi_naÃ¯ve_Treg | 22 |
| Tfh2 | NaÃ¯ve_Treg | 12 |
| Tfh2 | Gpr83+Igfbp4+_Treg_cells | 24 |
| Tfh2 | Tconv | 21 |
| Tfh2 | Helioshi_subpopulation | 40 |
| Tfh2 | Foxp3hi_subpopulation | 33 |
| Tfh2 | Lag3hi_subpopulation | 39 |
| Cd25hi_naÃ¯ve_Treg | Naive_B_cells | 21 |
| Cd25hi_naÃ¯ve_Treg | regulatory_B_cells | 9 |
| Cd25hi_naÃ¯ve_Treg | memory_B_cell | 17 |
| Cd25hi_naÃ¯ve_Treg | Somatic_hypermutation_B_cell | 29 |
| Cd25hi_naÃ¯ve_Treg | Plasma_cells | 29 |
| Cd25hi_naÃ¯ve_Treg | plasmablasts_cell | 8 |
| Cd25hi_naÃ¯ve_Treg | B10 | 35 |
| Cd25hi_naÃ¯ve_Treg | Memory_Tfh | 15 |
| Cd25hi_naÃ¯ve_Treg | Tfh17 | 22 |
| Cd25hi_naÃ¯ve_Treg | Tfh2 | 22 |
| Cd25hi_naÃ¯ve_Treg | Cd25hi_naÃ¯ve_Treg | 5 |
| Cd25hi_naÃ¯ve_Treg | NaÃ¯ve_Treg | 6 |
| Cd25hi_naÃ¯ve_Treg | Gpr83+Igfbp4+_Treg_cells | 12 |
| Cd25hi_naÃ¯ve_Treg | Tconv | 12 |
| Cd25hi_naÃ¯ve_Treg | Helioshi_subpopulation | 30 |
| Cd25hi_naÃ¯ve_Treg | Foxp3hi_subpopulation | 23 |
| Cd25hi_naÃ¯ve_Treg | Lag3hi_subpopulation | 27 |
| NaÃ¯ve_Treg | Naive_B_cells | 16 |
| NaÃ¯ve_Treg | regulatory_B_cells | 7 |
| NaÃ¯ve_Treg | memory_B_cell | 12 |
| NaÃ¯ve_Treg | Somatic_hypermutation_B_cell | 21 |
| NaÃ¯ve_Treg | Plasma_cells | 20 |
| NaÃ¯ve_Treg | plasmablasts_cell | 5 |
| NaÃ¯ve_Treg | B10 | 24 |
| NaÃ¯ve_Treg | Memory_Tfh | 6 |
| NaÃ¯ve_Treg | Tfh17 | 12 |
| NaÃ¯ve_Treg | Tfh2 | 12 |
| NaÃ¯ve_Treg | Cd25hi_naÃ¯ve_Treg | 6 |
| NaÃ¯ve_Treg | NaÃ¯ve_Treg | 2 |
| NaÃ¯ve_Treg | Gpr83+Igfbp4+_Treg_cells | 7 |
| NaÃ¯ve_Treg | Tconv | 8 |
| NaÃ¯ve_Treg | Helioshi_subpopulation | 17 |
| NaÃ¯ve_Treg | Foxp3hi_subpopulation | 15 |
| NaÃ¯ve_Treg | Lag3hi_subpopulation | 14 |
| Gpr83+Igfbp4+_Treg_cells | Naive_B_cells | 19 |
| Gpr83+Igfbp4+_Treg_cells | regulatory_B_cells | 6 |
| Gpr83+Igfbp4+_Treg_cells | memory_B_cell | 15 |
| Gpr83+Igfbp4+_Treg_cells | Somatic_hypermutation_B_cell | 26 |
| Gpr83+Igfbp4+_Treg_cells | Plasma_cells | 29 |
| Gpr83+Igfbp4+_Treg_cells | plasmablasts_cell | 9 |
| Gpr83+Igfbp4+_Treg_cells | B10 | 37 |
| Gpr83+Igfbp4+_Treg_cells | Memory_Tfh | 16 |
| Gpr83+Igfbp4+_Treg_cells | Tfh17 | 22 |
| Gpr83+Igfbp4+_Treg_cells | Tfh2 | 24 |
| Gpr83+Igfbp4+_Treg_cells | Cd25hi_naÃ¯ve_Treg | 12 |
| Gpr83+Igfbp4+_Treg_cells | NaÃ¯ve_Treg | 7 |
| Gpr83+Igfbp4+_Treg_cells | Gpr83+Igfbp4+_Treg_cells | 6 |
| Gpr83+Igfbp4+_Treg_cells | Tconv | 12 |
| Gpr83+Igfbp4+_Treg_cells | Helioshi_subpopulation | 30 |
| Gpr83+Igfbp4+_Treg_cells | Foxp3hi_subpopulation | 23 |
| Gpr83+Igfbp4+_Treg_cells | Lag3hi_subpopulation | 30 |
| Tconv | Naive_B_cells | 22 |
| Tconv | regulatory_B_cells | 8 |
| Tconv | memory_B_cell | 21 |
| Tconv | Somatic_hypermutation_B_cell | 26 |
| Tconv | Plasma_cells | 29 |
| Tconv | plasmablasts_cell | 12 |
| Tconv | B10 | 33 |
| Tconv | Memory_Tfh | 15 |
| Tconv | Tfh17 | 17 |
| Tconv | Tfh2 | 21 |
| Tconv | Cd25hi_naÃ¯ve_Treg | 12 |
| Tconv | NaÃ¯ve_Treg | 8 |
| Tconv | Gpr83+Igfbp4+_Treg_cells | 12 |
| Tconv | Tconv | 6 |
| Tconv | Helioshi_subpopulation | 28 |
| Tconv | Foxp3hi_subpopulation | 21 |
| Tconv | Lag3hi_subpopulation | 24 |
| Helioshi_subpopulation | Naive_B_cells | 25 |
| Helioshi_subpopulation | regulatory_B_cells | 12 |
| Helioshi_subpopulation | memory_B_cell | 34 |
| Helioshi_subpopulation | Somatic_hypermutation_B_cell | 36 |
| Helioshi_subpopulation | Plasma_cells | 58 |
| Helioshi_subpopulation | plasmablasts_cell | 24 |
| Helioshi_subpopulation | B10 | 61 |
| Helioshi_subpopulation | Memory_Tfh | 33 |
| Helioshi_subpopulation | Tfh17 | 42 |
| Helioshi_subpopulation | Tfh2 | 40 |
| Helioshi_subpopulation | Cd25hi_naÃ¯ve_Treg | 30 |
| Helioshi_subpopulation | NaÃ¯ve_Treg | 17 |
| Helioshi_subpopulation | Gpr83+Igfbp4+_Treg_cells | 30 |
| Helioshi_subpopulation | Tconv | 28 |
| Helioshi_subpopulation | Helioshi_subpopulation | 26 |
| Helioshi_subpopulation | Foxp3hi_subpopulation | 44 |
| Helioshi_subpopulation | Lag3hi_subpopulation | 52 |
| Foxp3hi_subpopulation | Naive_B_cells | 28 |
| Foxp3hi_subpopulation | regulatory_B_cells | 12 |
| Foxp3hi_subpopulation | memory_B_cell | 36 |
| Foxp3hi_subpopulation | Somatic_hypermutation_B_cell | 34 |
| Foxp3hi_subpopulation | Plasma_cells | 47 |
| Foxp3hi_subpopulation | plasmablasts_cell | 25 |
| Foxp3hi_subpopulation | B10 | 58 |
| Foxp3hi_subpopulation | Memory_Tfh | 26 |
| Foxp3hi_subpopulation | Tfh17 | 33 |
| Foxp3hi_subpopulation | Tfh2 | 33 |
| Foxp3hi_subpopulation | Cd25hi_naÃ¯ve_Treg | 23 |
| Foxp3hi_subpopulation | NaÃ¯ve_Treg | 15 |
| Foxp3hi_subpopulation | Gpr83+Igfbp4+_Treg_cells | 23 |
| Foxp3hi_subpopulation | Tconv | 21 |
| Foxp3hi_subpopulation | Helioshi_subpopulation | 44 |
| Foxp3hi_subpopulation | Foxp3hi_subpopulation | 24 |
| Foxp3hi_subpopulation | Lag3hi_subpopulation | 44 |
| Lag3hi_subpopulation | Naive_B_cells | 24 |
| Lag3hi_subpopulation | regulatory_B_cells | 12 |
| Lag3hi_subpopulation | memory_B_cell | 36 |
| Lag3hi_subpopulation | Somatic_hypermutation_B_cell | 36 |
| Lag3hi_subpopulation | Plasma_cells | 54 |
| Lag3hi_subpopulation | plasmablasts_cell | 21 |
| Lag3hi_subpopulation | B10 | 60 |
| Lag3hi_subpopulation | Memory_Tfh | 33 |
| Lag3hi_subpopulation | Tfh17 | 40 |
| Lag3hi_subpopulation | Tfh2 | 39 |
| Lag3hi_subpopulation | Cd25hi_naÃ¯ve_Treg | 27 |
| Lag3hi_subpopulation | NaÃ¯ve_Treg | 14 |
| Lag3hi_subpopulation | Gpr83+Igfbp4+_Treg_cells | 30 |
| Lag3hi_subpopulation | Tconv | 24 |
| Lag3hi_subpopulation | Helioshi_subpopulation | 52 |
| Lag3hi_subpopulation | Foxp3hi_subpopulation | 44 |
| Lag3hi_subpopulation | Lag3hi_subpopulation | 24 |
